# How did the guppy Y chromosome evolve?

**DOI:** 10.1371/journal.pgen.1009704

**Published:** 2021-08-09

**Authors:** Deborah Charlesworth, Roberta Bergero, Chay Graham, Jim Gardner, Karen Keegan

**Affiliations:** 1 Institute of Evolutionary Biology, School of Biological Sciences, University of Edinburgh, Edinburgh, United Kingdom; 2 University of Cambridge, Department of Biochemistry, Sanger Building, 80 Tennis Court Road, Cambridge, United Kingdom; University of Georgia, UNITED STATES

## Abstract

The sex chromosome pairs of many species do not undergo genetic recombination, unlike the autosomes. It has been proposed that the suppressed recombination results from natural selection favouring close linkage between sex-determining genes and mutations on this chromosome with advantages in one sex, but disadvantages in the other (these are called sexually antagonistic mutations). No example of such selection leading to suppressed recombination has been described, but populations of the guppy display sexually antagonistic mutations (affecting male coloration), and would be expected to evolve suppressed recombination. In extant close relatives of the guppy, the Y chromosomes have suppressed recombination, and have lost all the genes present on the X (this is called genetic degeneration). However, the guppy Y occasionally recombines with its X, despite carrying sexually antagonistic mutations. We describe evidence that a new Y evolved recently in the guppy, from an X chromosome like that in these relatives, replacing the old, degenerated Y, and explaining why the guppy pair still recombine. The male coloration factors probably arose after the new Y evolved, and have already evolved expression that is confined to males, a different way to avoid the conflict between the sexes.

## Author summary

We report new findings concerning the long-studied the guppy XY pair, which has remained somewhat mystifying. We show that it can be understood as a case of a recent sex chromosome turnover event in which an older, highly degenerated Y chromosome was lost, and creation of a new sex chromosome from the ancestral X. This chromosome acquired a male-determining factor, possibly by a mutation in (or a duplication of) a previously X-linked gene, or (less likely) by movement of an ancestral Y-linked maleness factor onto the X. We relate the findings to theoretical models of such events, and argue that the proposed change was free from factors thought to impede such turnovers. The change resulted in the intriguing situation where the X chromosome is old and the Y is much younger, and we discuss some other species where a similar change seems likely to have occurred.

## Introduction

Despite having been the object of study for more than 100 years, and being the first example of a Y chromosome that carries genes [1], the guppy sex chromosome pair remains puzzling. The chromosome that carries the male-determining locus is only minimally differentiated from its X chromosome counterpart [2], and genome sequencing has revealed that all genes appear to be present on both of the homologues, with similar depth of coverage [3,4]. This chromosome, the homologue of chromosome 12 of the medaka [5,6], is called LG12. Genome sequencing, by both Illumina short-read [3,4,7] and Pac-Bio long-read approaches [6], has yielded no evidence for a non-recombining region with consistent Y-specific sequence variants or features. Cytogenetic studies, however, detected a greater length of the Y than the X chromosome, and an LG12 region with male-specific heterochromatin and male-specific repetitive sequences [8–10], which is thus suspected to include the male-determining factor. This factor was recently located genetically to a chromosome 12 region similar to that identified cytologically [11].

The lack of Y-X sequence differentiation suggests that the male-determining locus may be physically small, with only a small closely linked differentiated region, and the absence of any region of low coverage depth in males also suggests ongoing recombination, preventing genetic degeneration, consistent with viability of homozygotes for Y chromosomes with different origins [12], and with detection of occasionally crossovers between the Y and X chromosomes [13–15]. Analyses of genome sequences support the conclusion that most of the chromosome pair recombine, though two very different recombining regions are defined by genetic mapping in males. The tip of the 26.5 megabase (Mb) XY pair is a physically small pseudo-autosomal region (which we term “PAR1” [11]) like those in mammal sex chromosome pairs, which recombine at a very high rate in male meiosis, probably because a crossover is regularly located within this region [16]; this terminal PAR1 region occupies less than 2 Mb of the 26 or more megabases of chromosome 12 [3,11]. In contrast, the other recombining region (PAR2), extending across most of the chromosome, from the centromere end to at least 20 Mb, has a much lower rate of recombination per Mb in male than female meiosis [3]. As guppy autosomes show a similar crossover localisation to the chromosome termini in male meiosis [3], the pattern on LG12 is probably genome-wide, and may not have evolved because this chromosome carries the sex-determining locus [17].

In contrast, the homologous Y chromosomes in the closely related species, *Micropoecilia picta* and *M*. *parae*, are highly degenerated [18,19], which suggests that chromosome 12 has been a sex chromosome for a long evolutionary time, though not necessarily with the same male-determining factor. This observation of an old-established, degenerated Y raises the question of how the guppy can have an undegenerated Y chromosome. Specifically, theoretical models (see the Discussion section) predict that presence of a degenerated Y chromosome prevents “turnover” events in which a new sex-determining factor arises and spreads in a species, replacing its ancestral sex-determiner.

**Fig 1:**
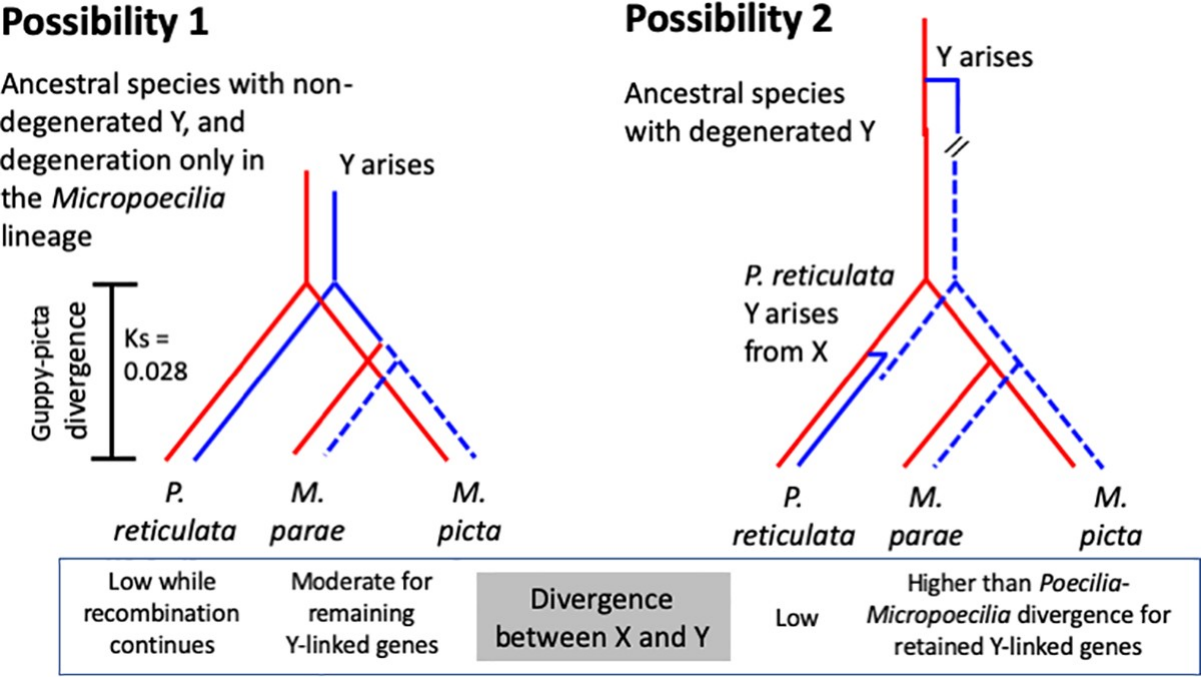
The two possible evolutionary histories of the guppy and *Micropoecilia* Y chromosomes. The diagrams show the possibilities for the ancestry of the two *Micropoecilia* species whose Y chromosomes have degenerated (symbolized by dashed blue lines), and the split of the *Poecilia* and *Micropoecilia* lineages. The expected differences that might allow the two possibilities to be distinguished are shown below the diagrams (see text for explanations).

This prediction would suggest that LG12 has persisted as a recombining chromosome pair over its past evolutionary history, preventing genetic degeneration and confining Y-X differentiation to a physically small genome region surrounding the male-determining locus, and that the guppy retains this state, whereas differentiation proceeded rapidly in *Micropoecilia*, for unknown reasons. This scenario (possibility 1 in Fig 1) was previously proposed [7,19]. A different possibility (2 in Fig 1), is that the two sex chromosomes of the guppy have very different evolutionary ages: its X is an old-established X chromosome, but its Y evolved recently, in a turnover event, and complete recombination suppression has not evolved [18]. The much greater sex chromosome differentiation in *Micropoecilia* need not indicate the proposed much slower rate of differentiation of the guppy Y than in *Micropoecilia* [7,19]. It may simply reflect the longer time since the evolution of complete recombination suppression (which may have occurred either in *Micropoecilia*, or in an ancestor before the split from the guppy). As discussed later, this previously unsuspected type of turnover is not prevented by the presence of a degenerated Y chromosome.

Long-term maintenance of a physically limited sex-determining region like that in the guppy, in a sex chromosome pair that failed to evolve suppressed recombination (possibility 1), cannot be ruled out *a priori*, though we are not aware of any other plausible example. Such a scenario in male guppies appears to conflict with the expectation that sexually antagonistic (SA) phenotypic effects of male coloration polymorphisms should have selected for close linkage with the male-determining locus [20]. However, SA coloration factors in guppies (and possibly other SA factors) could be over-represented on the guppy Y because SA mutations are most likely to spread in a population if they occur in genes that are closely linked to the sex-determining gene [21], as close linkage allows alleles that benefit males to maintain associations with the male-determining allele. If linkage is close, selection for even closer linkage with the sex-determining gene will be weak. Moreover, evolution of male-limited expression can eliminate selection for closer linkage. Consistent with their having initially had SA effects, guppy male coloration factors are predominantly Y linked, but a few have been found on the X, either because of occasional recombination [14], probably in the PAR2 region, or because they are within the PAR1 region. Such partially Y linked factors are not expressed when they are carried by females, as reviewed in ref. [22]. Male-limited expression, rather than closer linkage to the sex-determining gene, might therefore have evolved.

Finally, although the low recombination rate across most of LG12 in the guppy appears consistent with the SA polymorphism theory for recombination suppression between Y and X chromosomes, there is no evidence that linkage with the male-determining locus has become closer than before the SA polymorphisms were established. The alternative is that the crossover pattern in male meiosis, with strong localisation to the chromosome termini, might already have been established in an ancestral population, and retained in extant guppies and perhaps related species [3,17].

It is therefore worthwhile to consider possibility 2 in Fig 1: that the guppy Y arose in a species whose Y chromosome was already highly degenerated, like the LG12 Y in *M*. *picta* and *M*. *parae*, which have lost an estimated 99% of the genes present on the X [7,18,19]. A turnover event in the *Poecilia* lineage, before the very recent split between *P*. *reticulata* and *P*. *wingei*, could have led to replacement of a degenerated Y by a new Y that is very similar to the *Micropoecilia* X. Synonymous site divergence between *P*. *wingei* and *P*. *reticulata* is less than 2%, based on a set of 5 genes (S1A Fig), and the change could have occurred in a common ancestor. Because of strong crossover localisation in guppy male meiosis, the appearance of a male-determining factor could have initiated largely male-specific transmission of the chromosome [3].

Here, we describe tests of the hypotheses in Fig 1. Ideally, one should estimate sequence divergence between fully Y- and X-linked genes in at least one of the *Micropoecilia* species. Under possibility 2, this divergence will be large, reflecting the long time since recombination between them stopped (contrasting with low divergence between the Y and X sequences in the guppy, see Fig 1, or in *P*. *wingei*). Y-X divergence should also exceed the divergence of X-linked sequences from their orthologues in the guppy or *P*. *wingei*. However, our intensive efforts lead to the conclusion that the *M*. *picta* Y-linked region carries no genes homologous to genes on the X (see Results below). We were therefore unable to estimate Y-X divergence. Instead, we estimated divergence between orthologues in the guppy and *M*. *picta*, and discuss whether there has been enough time for the highly genetically degenerated state of the *Micropoecilia* Y to evolve since the split from the lineage that includes the guppy and *P*. *wingei*, as possibility 2 proposes. Loss of almost all genes, and the subsequent *de novo* evolution of dosage compensation in the *Micropoecilia* lineage [19], take considerable amounts of evolutionary time [23].

## Results

### Analysis of coverage in M. picta and M. parae males and females, and search for genes with diploid coverage in M. picta males

Microsatellite genotyping indicated hemizygosity of chromosome 12 for all loci tested, in both *M*. *picta* and *M*. *parae* [18]. Hemizygosity was confirmed for *M*. *picta* by coverage data obtained by high-throughput sequencing approach (SeqSNP, see Methods). The full details are in a S1 Methods file. The SeqSNP data yielded an estimate of 99% gene loss from the Y [18].

**Fig 2:**
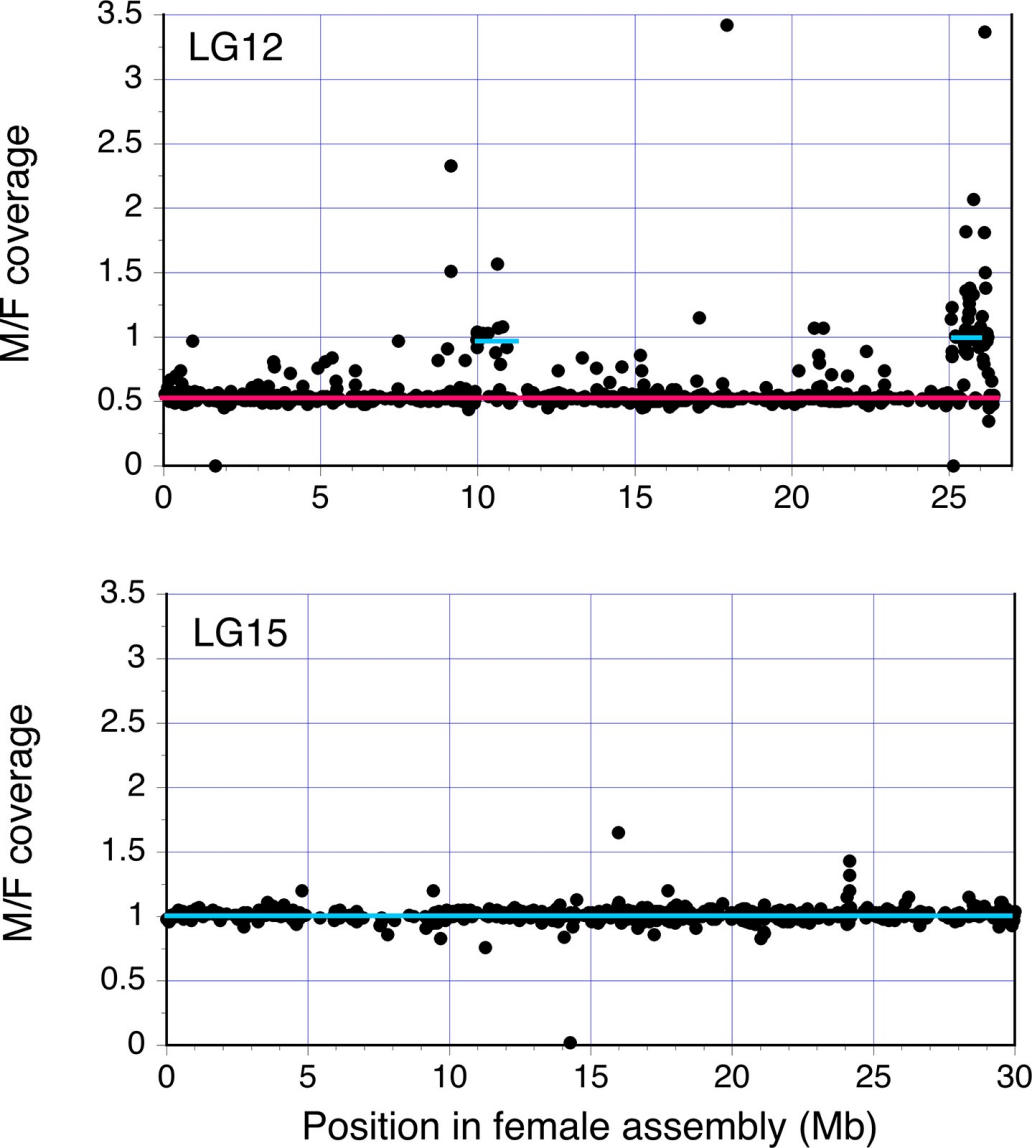
Coverage depths of genes on the *M*. *picta* sex chromosome and an autosome. In each part, the y axis shows estimated normalized coverage values and the x axis shows physical positions in the guppy reference assembly of the homologous chromosome. In both the guppy and *M*. *picta*, chromosome 12 is the sex chromosome, and LG15 is shown as a representative autosome (as explained in the text, other autosomes are similar). The red horizontal line indicates a value of 0.5, as expected for hemizygous genes, and the blue one indicates diploid coverage (a value of 1), as seen for most genes across the autosome, and for two clusters of LG12 genes.

Our new analyses of coverage, used whole genome low coverage sequences of multiple males and females sampled from multiple natural populations. The Methods sub-sections “Estimating divergence between *Poecilia* and *Micropoecilia* sequences” and “Analysis of coverage in *M*. *picta* males and females”, describe our reference-guided local assembly approach to map *M*. *picta* sequences to genes in the *P*. *reticulata* female reference genome sequence [5]. The analysis included many more genes than SeqSNP, and confirmed that most guppy LG12 genes show haploid coverage in *M*. *picta* males, while autosomal genes show diploid coverage levels. Fig 2 shows the normalised coverage male/female (M/F) ratios in the two sexes for chromosome 12 and, for comparison, an autosome (LG15). The ratios for the autosomal genes were indeed consistently close to 1 for the vast majority of the 824 LG15 genes where this could be estimated (the median value was 1.01 and only 5 genes yielded values below 0.7). Other autosomes gave very similar results (see Dryad accession number https://doi.org/10.5061/dryad.cjsxksn65); the results are similar to those already published for *M*. *picta* [19]. For LG12, the ratios are close to 0.5, indicating hemizygosity, for 822 genes whose orthologue positions in the guppy assembly span most of the chromosome (median 0.53). There appear to be slightly more outliers for chromosome 12 than for the autosome, possibly reflecting transposable element or other repetitive sequences; however, as the sequences analysed were genes, there should be few such cases.

Male hemizygosity for LG12 genes appears to be species-wide in both *M*. *picta*, including in our *M*. *picta* males from Trinidad samples where *M*. *parae* is absent, as well as in all *M*. *parae* populations tested [19,24,25]. We found the same state in a third *Micropoecilia* species, *M*. *bifurca*, based on genotypes of six informative microsatellite markers spanning most of the sex chromosome (S6 Table).

We next searched the *M*. *picta* LG12 gene coverage results for any genes that might be retained on the Y chromosome of this species, using the same natural population samples. Genetic degeneration is expected to produce consistent signals of hemizygosity across all populations of a species, and lack of degeneration should consistently yield similar depths of coverage in both sexes (see Methods and S3A Fig). Sequences with male-female coverage ratios close to 1 could represent either PAR genes in *M*. *picta*, or genuinely non-hemizygous parts of the fully sex-linked region (so called “XY” sequences), or autosomal sequences that have been mis-assembled on the guppy LG12. This will affect our assemblies, as the guppy genome assemblies were used to assign positions for our assembly of the *M*. *picta* sequences.

In contrast to the majority of LG12 genes described above, 47 of 57 genes with positions distal to 25 Mb in the guppy female assembly (from 25,067,709 to 26,240,873 bp) had M/F coverage ratios close to 1 (the median value for these 57 genes = 1.0); their terminal locations in the guppy genome assemblies suggest that these might be PAR genes in both species. In addition, 16 of the 21 genes with positions from 9,954,185 to 10,927,843 bp in the female guppy assembly, also had high values (the median for this set was 0.95). The values for these sets both differed significantly from the ratios of genes in the hemizygous regions, though 12 genes at the terminal end of the guppy assembly (26,262,815 to 26, 423,538 bp) had lower values (median 0.53). These may represent an error in the guppy assembly, as their genetic map positions are not consistent with the positions assigned in the female guppy assembly [11], and the positions have been changed in the male assembly, based on PacBio sequencing long reads, which also does not support an inverted order of the X or the Y [6]. It seems likely that these may not to be PAR genes, though definitive evidence will require genetic mapping with denser genetic markers. Given their locations and diploid coverage values in males, the set of 47 terminal region genes seem likely to be PAR genes in *M*. *picta*. In the next section, we describe genetic mapping results supporting this conclusion.

Before doing so, we evaluate the set of 16 genes that are assigned positions near 9–10 Mb in the guppy female assembly. As noted above, these might be autosomal genes that have been mis-assembled, or further PAR genes, rather than fully Y-linked in *M*. *picta*, but not hemizygous (so called “XY” sequences). Several observations support the mis-assembly hypothesis. First, these genes’ positions in the guppy female assembly are not well supported. This region is repetitive, and is much more AT-rich than its neighbouring regions in the guppy female assembly (S4 Fig), and the gene sequences have low mapping quality. Second, consistent with assembly problems, these sequences are placed in a different location in the guppy male assembly, where they appear in a centromere-proximal region, between 2. 6 and 3.1 Mb [6], but they map near the end of the platyfish (*Xiphophorus maculatus*) assembly that corresponds with the terminal, pseudo-autosomal, part of the guppy assembly (S5 Fig). Comparison with the platyfish assembly finds a set of sequences from this LG12 region (including several genes, and a repetitive sequence) on Xm1, the homologue of the guppy autosome, LG7 (S6 Fig). An LG7 location is also supported by the observation that a single long contig in our *M*. *picta* whole genome sequence [26] carries these genes along with homologues of many guppy LG7 genes. Overall, therefore, our detailed coverage analyses did not detect any good candidates for *M*. *picta* genes with retained Y-linked alleles.

Unlike the hemizygous genes, which gave consistent signs of hemizygosity in most populations, genes that appeared to be duplicated were mostly detected only in samples from individual populations (S3B Fig). At most, 32 genes suggested consistent evidence from all populations, and these were not studied further. We did, however, perform a further test for genes in the terminal part of LG12 that might have Y-linked or other duplications, using BLAT searches of *M*. *picta* contigs with all genes in the guppy PAR and adjacent regions as the queries, and excluding targets that were not single-copy in *M*. *picta*. The few gaps in the region distal to 20 Mb tend to correspond with regions of low gene density, and to gaps between contigs, and do not indicate duplicated genes in the most terminal region, with the highest M/F ratios (S7 Fig).

Moreover, we note that the coverage ratios in the putative PAR are not all due to coverage in males exceeding that for autosomal genes, as Fig 2 might suggest, but frequently reflect lower coverage in females, relative to autosomal genes. S8 Fig shows these results for the part of the *M*. *picta* LG12 corresponding to the region shown in S7 Fig, relative to the values for an autosome, LG15. Although we excluded low coverage and low-quality sequences (as described in S1 Methods 1), genes in the region between 20 and 24 Mb (where the M/F coverage ratio remains close to 0.5), have low relative (M/A and F/A) values than for the rest of LG12. This suggests possible problems with mapping reads in this region. In the 64 genes in the most distal region, where M/F coverage values are often, only ten genes have higher relative coverage in males than females, 14 have similar coverage in both sexes, and 40 have lower values in males, and the overall median M/F ratio is 0.98. These results suggest highly variable success in mapping reads from PAR sequences, perhaps due to their high GC content [17].

### Genetic tests of sex linkage in the guppy and M. picta

The observation that coverage is similar in males and females for most *M*. *picta* homologues of guppy PAR genes suggests that the *M*. *picta* sex chromosome pair has not evolved into a X0 system, but retains a small PAR region carrying genes homologous to those in the guppy PAR. Alternatively, this region might no longer be part of the *M*. *picta* sex chromosome, i.e. these genes might be autosomal in this species.

To test whether these genes are sex-linked or autosomal in this species, and in *M*. *parae*, we studied two genes from the terminal region of the guppy sex chromosome, *zer1* and *fpgs* (see Methods); in the guppy genome assembly, both are distal to the most distal fully sex-linked marker genetically mapped [3]. We next describe results demonstrating that both genes show sex-linkage, and must on be located on LG12 in all three species. To distinguish between complete and partial sex-linkage, we also genotyped samples from natural populations.

Linkage analysis of the *zer1* gene in the guppy QHPG5family (see Methods), based on length differences of intron 10 of this gene, confirms that this gene is partially sex-linked in the guppy. It recombines with the sex-determining locus in male meiosis, and its genetic map location is between two other pseudoautosomal markers CTT98 and GT354 (S7 Table of ref. [18]), also included in the Dryad files for the present manuscript). The *fpgs* gene did not have markers suitable for mapping in the guppy.

In *M*. *parae*, variants in families suggest sex linkage of the *fpgs* gene, though the family sizes are small. The *zer1* gene was not polymorphic in this species and therefore could not be mapped (S5 Table of ref. [18]). Genotypes in natural populations, however, suggest that both these genes appear to be partially sex-linked, as in the guppy (S4 and S5 Table). Among 24 individuals genotyped for *fpgs* intron 4 length variants, seven of the 13 males were heterozygous, and two alleles were shared with the female sample. Three males were heterozygous for an allele not seen among the females, but only 5 females were present in the sample, so these are probably merely low frequency alleles, not male-specific. Similarly, many males were heterozygous for length variants of the *zer1* gene intron 10 shared with females (in total, 5 different alleles were detected in this small sample of males, two of which, plus one other allele, were found in the small female sample).

In *M*. *picta*, the *zer1* gene is sex-linked in families from Trinidad and Suriname rivers, based on variable intron 10 sizes. In all families whose sires were not homozygous for the allele with length 957-bp, only the male progeny inherited the father’s other allele (families T1, T2, T6, T7 and S3 in S4 Table). However, genotypes in samples from natural populations suggest complete sex linkage (S5 Table). All 31 Trinidad females were homozygous for a 957 bp allele of *zer1* (also present in the Suriname sample, but not fixed there). However, among 31 Trinidad males, only 18 were homozygous for this allele; the other 13 were heterozygous for one of at least eight male-specific alleles, suggesting that *zer1* does not recombine with this species’ sex-determining locus in male meiosis. The 957-bp allele thus appears to be carried by all X chromosomes, while the Y is highly polymorphic for many different Y-specific alleles. SNPs in the *fpgs* gene also show sex-linkage in male meiosis (family S3 in S4 Table). In the Trinidad natural population samples, all males appeared to be homozygotes for the microsatellite marker in this gene, including two males with rare variants, one of which was also present in a female heterozygote, suggesting that males are hemizygous, and that this gene is also completely sex linked in *M*. *picta* (S5 Table). The broods from all seven Trinidad females also all appeared to be sired by homozygotes; however, two Suriname sires were inferred from their progeny genotypes to be heterozygous for the SNP marker in the gene, and therefore this region cannot be hemizygous (S4 Table). Overall, the data suggest that the *zer1* gene, and possibly the more distal *fpgs* gene, are completely sex-linked in *M*. *picta*, but partially sex-linked in the guppy and *M*. *parae* (Table 1). Some apparently hemizygous males might simply reflect heterozygtes for common null alleles for the *fpgs* gene microsatellite. The *M*. *picta* Y chromosome may therefore have stopped crossing over between the XY pair in a region that still recombines in the other two species.

**Table 1:** Results of genetic mapping or genotyping of two guppy PAR genes in two *Micropoecilia* species.

Gene	Position in female guppy assembly	Sex linkage		
		Guppy	*P*. *picta*	*P*. *parae*
*zer1*	26,208,835	Partial	Complete, but not hemizygous	Partial
*fpgs*	26,286,204	Partial	Possibly complete, but not wholly hemizygous	Partial

### Sequence divergence between Poecilia and Micropoecilia species and the time when genetic degeneration occurred in Micropoecilia

**Fig 3:**
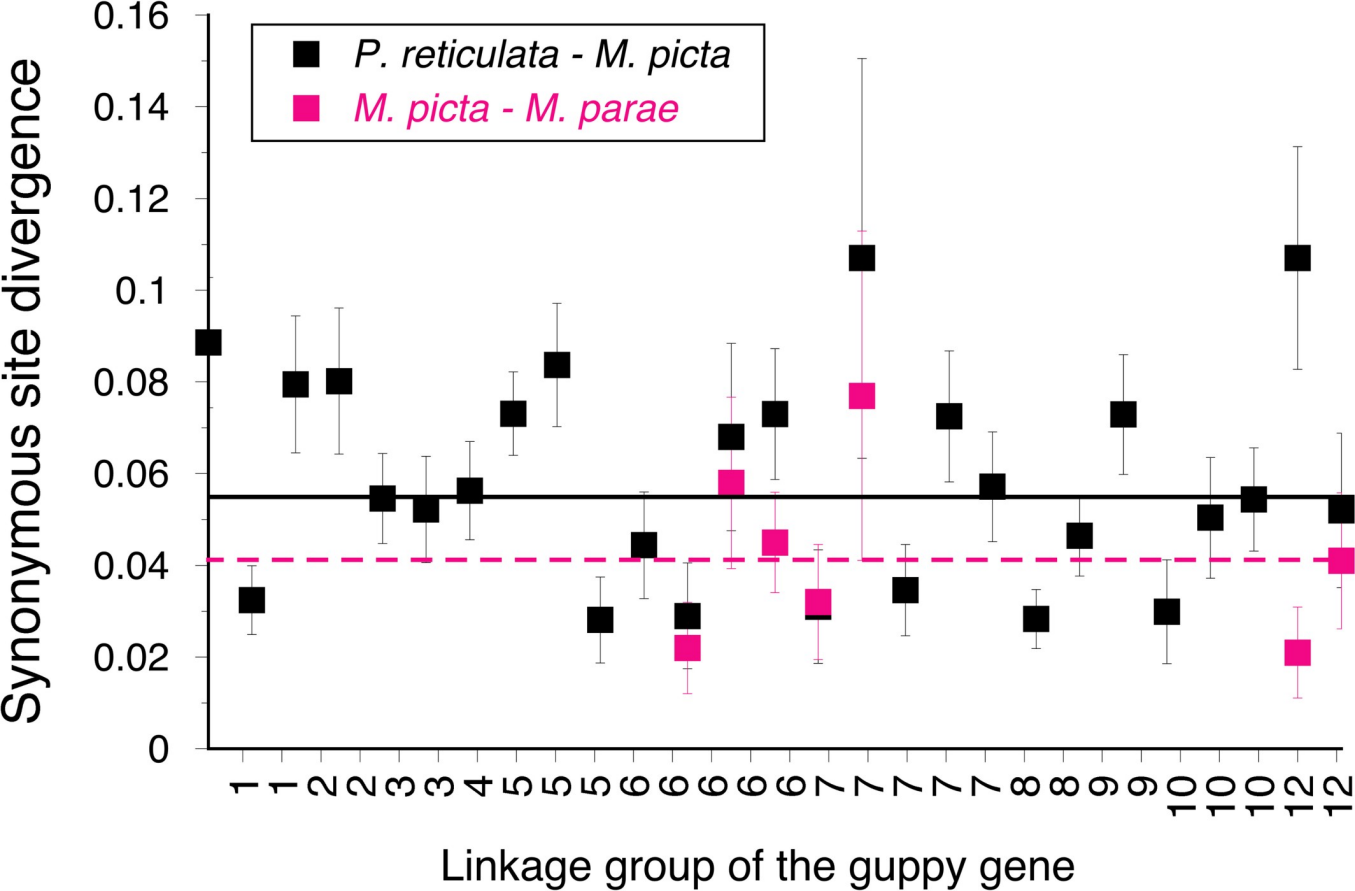
DNA sequence divergence between the guppy and *M*. *picta* and between two *Micropoecilia* species. Divergence estimates are for synonymous sites in coding regions of 27 genes, with Jukes-Cantor correction for saturation of substitution, and 95% confidence intervals of the estimates. The horizontal lines indicate the median values (5.4% for *P*. *reticulata* versus *M*. *picta*, and 4.1% for *M*. *picta* versus *M*. *parae*. Divergence estimates between all three *Micropoecilia* species are given in S3B Table.

Finally, we wished to test between the two possible evolutionary origins of the guppy Y chromosome outlined in the Introduction section above. Because no genes seem likely to be Y-linked copies that have escaped loss during the degeneration in *M*. *picta* Y chromosome, we cannot compare the time of Y-X divergence with the time since the split of *M*. *picta* and *M*. *parae* from the lineage leading to *P*. *reticulata*. However, we have estimated synonymous site divergence (*K*_s_ values, see Fig 3) between *M*. *picta* and *P*. *reticulata* for 27 genes, from several different chromosomes (see Methods). The median *K*_s_ value is 0.055. If the *M*. *picta* Y stopped recombining and started to degenerate after the split of the two lineages (possibility 1 of Fig 1), its complete degeneration, and evolution of equal expression of sex-linked genes in males and females, indicating dosage compensation [19], must have occurred in an evolutionary time corresponding to at most half this value, or a *K*_s_ value of about 2.8%. Given that our microsatellite genotyping suggests that the *M*. *parae* and *M*. *bifurca* Ys are also highly degenerated (see results above and ref. [24]), and that synonymous site divergence between the *Micropoecilia* species indicates a split at least half as long ago as the split between the *Poecilia* and *Micropoecilia* lineages (as illustrated in Fig 3), it is likely that the degeneration occurred in the *Micropoecilia* lineage before it split from the *Poecilia* lineage, rather than after the *Micropoecilia* species split. This conclusion is conservative, as the only other possibility if the ancestor had a non-degenerated Y (possibility 1 above) is that degeneration occurred in one *Micropoecilia* species, and the degenerated Y introgressed into the other species. This implies complete degeneration during a time when the three species were able to hybridise, most likely early in the time after the split from the *Poecilia* lineage. In the next section, we assess whether the time is long enough for complete genetic degeneration to evolve.

## Discussion

### How degenerated is the M. picta Y-linked region?

The evidence described above confirms that the *M*. *picta* Y-linked region is highly degenerated. Our analysis suggest that the Y carries at most 60 genes, compared with about 900 on the X, and that most of these are orthologous to genes at the terminal (PAR) end of the guppy chromosome 12 in either the female or male assembly.

It is not yet completely excluded that the *M*. *picta* Y chromosome might carry sequences similar to their X-linked sequences, with the Y-linked ones too diverged to be detected by previous analyses [7,18,19]. Highly diverged Y-linked gene sequences could fail to map to the relevant location on the *P*. *reticulata* reference X. To maximise our ability to detect any Y-linked sequences that are still present, our analyses, high-throughput genotyping with primers designed from *P*. *reticulata* [18], and mapping to genome sequences, described here, targeted coding sequences, which are least likely to diverge. The mean divergence of *M*. *picta* sequences from guppy sequences for all site types, which is the most relevant for the success or failure of PCR, is only 2.7% (see S3 Table), and indeed most targeted genes in *M*. *picta* were successfully amplified in our high-throughput genotyping experiment. Any undetected gene sequences retained on the Y of this species must be more diverged than this average.

Our coverage analysis using genome sequences of males should have detected some of these diverged sequences, if they indeed exist. The read mapping employed was very sensitive, but was quite intolerant of mismatches, and would therefore be unlikely to call Y-linked sequences that are extremely diverged. Genes whose X cognates are missing from the reference X (notably in difficult to assemble, repetitive regions) would also remain undetected. However, despite employing a *P*. *reticulata* reference sequence, our mapping had a high overall recall value at high quality (for the 13 females from the CAR_H population alone, we recovered 140 out of an initial original 180.5 million reads, or 78%, see S1 Methods 1). This conservative statistic suggests that only small amount of raw sequence was too diverged to map. The larger number of genomes used for the coverage analysis included much higher numbers of reads, and recall was similar for both sexes. A better assembly of *M*. *picta* in the future should indicate whether some regions have so far escaped inclusion in assemblies, and might yield an even higher recall value, and further reduce the proportion of unmapped sequence. Our detailed analyses of LG12 detect fewer than 100 genes, many of which are probably PAR genes. This is consistent with the inferred number of guppy PAR genes, based on genetic mapping of genes with known physical positions in the assemblies [3], or on high GC content of their introns [17]. As described above, PAR genes are also present in *M*. *parae*. Neither *Micropoecilia* species appears to have an X0 system.

Under the proposed scenario in which the guppy Y evolved from an X chromosome (possibility 2), the boundary of the highly recombining PAR1 of chromosome 12 might differ from the PAR boundary in *Micropoecilia* species. The evidence from the *zer1* gene (see Results) suggests that this boundary might indeed differ, and that the *M*. *picta* PAR could be slightly smaller than the guppy one. Further data are needed to determine the PAR boundaries in both species, and to test whether any difference reflects a change in *M*. *picta*.

Overall, we conclude that (apart from PAR genes) the *M*. *picta* Y has probably retained at most a small proportion of coding sequences ancestrally present on the X. To estimate the extent of gene loss from the *M*. *picta* Y since it stopped recombining with the X chromosome, we can assume that the guppy and *M*. *picta* X chromosomes carry similar numbers of genes, as our results show that the *M*. *picta* PAR does not include many more genes than the terminal guppy PAR1. On this basis, the X-linked region carried about 800 ancestral genes when recombination stopped in the *Micropoecilia* lineage, of which at most 16, or 2%, have been retained on the Y.

Both our results [18] and genome sequencing [24] suggest that Y chromosome degeneration is also advanced in *M*. *parae*. The finding of male hemizygosity in all three *Micropoecilia* species tested (see Results and S6 Table), supports the suggestion that degeneration probably evolved in a common ancestor before these species split [24]. The variant version of possibility 1 (outlined above), involving introgression of a degenerated Y into two other species, is therefore not likely.

More specifically, recombination probably ceased in a common ancestor, though degeneration might have proceeded independently in the three *Micropoecilia* lineages. As microsatellite sequences are in non-coding regions and introns, the hemizygosity of LG12 microsatellites in males suggests that parts of this chromosome have been deleted and that only a remnant of the ancestral Y remains. This conclusion remains to be tested by cytogenetic observations of the physical size of the *M*. *picta* and *M*. *parae* Ys.

### How long has the M. picta Y been non-recombining, and is it long enough for complete genetic degeneration?

The lack of any good candidate genes with both X- and Y-linked copies described above prevents our estimating Y-X sequence divergence to understand whether the *M*. *picta* Y stopped recombining with the X chromosome before the split from the *Poecilia* lineage, and, if so, how long before. However, it is still possible to evaluate the two possibilities for the origin of the guppy sex chromosomes summarised in Fig 1, because, under possibility 1, degeneration in the *Micropoecila* species started after their split from *Poecilia* lineages (Fig 1A). We can therefore assess whether there was enough time for almost complete degeneration to occur. The *K*_s_ value of 2.7% mentioned above over-estimates the time of the split, because it is a “raw divergence” value that includes an unknown contribution from polymorphisms within the ancestral species [27]. The synonymous site diversity in the ancestor is unknown, and there are as yet no estimates from the species of interest, so we cannot currently correct for such polymorphism and estimate the net divergence value that estimates the separation time in terms of fixed substitutions since the time of the split [27]. However, even conservatively using the raw divergence value, it seems unlikely to represent enough time for the documented almost complete gene loss from the fully Y-linked region, and evolution of dosage compensation [19]. Given that all three *Micropoecilia* species so far studied appear to have degenerated Y chromosomes, it is unlikely that the Y chromosome stopped recombining in a common ancestor and degeneration occurred independently in each lineage after their split. Based on synonymous site divergence estimates (S3B Table), this restricts the available time to about half that suggested by the time of the guppy-*M*. *picta* split.

The evolutionary time needed for such changes is not yet fully understood, but we next outline current predictions and empirical information from animals (data from plants are less helpful, because selection in the haploid pollen may cause degeneration to be slow, as reviewed in ref. [28]).

Empirical data are very scarce on the relationship between the extent of degeneration, and divergence at synonymous sites (which, assuming a rough molecular clock, relates well to the number of generations since recombination stopped). The best data are from humans. However, all regions of the human Y have become highly degenerated, with the Y-linked genes either being completely lost, or present but non-functional [29], including the two young strata in humans that are pseudo-autosomal in other mammals, including a lemur species [30,31]. These two strata are not very young, as they are present in lemur species [32], and have mean Y-X *K*_s_ values of 45% and 14% (the estimates are rough, because these strata include only 17 X-linked genes [29]). The human strata are already too old to provide information about partial degeneration over times corresponding to that for the *Micropoecilia* lineage degeneration.

The currently available data of which we are aware for degeneration over smaller evolutionary times are listed in S7 Table. Although data are scanty, all species with Y-X (or W-Z) *K*_s_ > 20% show complete degeneration, but degeneration is incomplete when *K*_s_ < 20%, including the very well-studied *D*. *miranda* neo-Y chromosome [33]. Data are available for a few fish fully sex-linked regions, and these are incompletely degenerated, despite having *K*_s_ considerably higher than the value corresponding to the time available in *Micropoecilia*. The oldest stratum in the threespine stickleback, *Gasterosteus aculeatus* (with about 600 genes, slightly fewer than in the species analysed here), has an estimated X-Y *K*_s_ = 16% (or a time back to the Y-X split corresponding to *K*_s_ = 8%, assuming the same neutral substitution rate for both X- and Y-linked regions) yet 18% of X-linked genes still have detectable Y-linked copies. This fully sex-linked region is shared with *G*. *wheatlandi*, but in that species only 8% of genes are found on the Y, and these yielded a lower *K*_s_ estimate, 9% [34]. In both species, many Y-linked genes are still present in regions that stopped recombining more recently; in G. *aculeatus*, regions with Y-X *K*_s_ = 3–4% (corresponding to an evolutionary time similar to that available for degeneration of the *Micropoecilia* Y) have retained around 70% of genes [35]. Even during double this time, gene loss might conservatively be expected to be at most 60% (double the 30% value estimated for the stickleback), given that initial rapid degeneration quickly slows down as the number of remaining functional genes undergoing deleterious mutations declines [23]. In an unrelated fish, *C*. *semilaevis*, 44% of fully sex-linked genes are present on the W as well as the Z, indicating much less degeneration than in *M*. *picta*, but the W-Z divergence time is considerably longer, with W-Z *K*_s_ estimated to be 15% [36]. The time available therefore seems unlikely to be compatible with the almost complete gene loss from the *M*. *picta* Y, especially if the degeneration occurred before the split from other *Micropoecilia* species, as argued above.

Theoretical modelling provides another source of information. However, the time needed for complete degeneration of a Y chromosome cannot yet be predicted, and many uncertainties remain. One set of parameter values modelled led to 90% degeneration of a Y-linked region with 2,000 genes in about 5 million generations, but with < 1,000 genes degeneration is greatly slowed (compare the right-hand panels of Figs 8 and 4 of ref. [23]).The *K*_s_ value of 0.054 between the guppy and *M*. *picta* estimated above suggests about 27 million generations since the *Micopoecilia* species split from the *Poecilia* lineage (assuming a neutral mutation rate of 10^−9^ per site per generation), which might be enough time for nearly complete degeneration, given about 800 ancestral genes (see above). Degeneration can be faster with smaller population sizes, higher deleterious mutation rates, stronger selection against deleterious mutations, or unequal fitness effects of strongly deleterious mutations [23], but allowing mutations on the X in a diploid population can slow it down [37]. The genetic degeneration process initially involves Muller’s ratchet combined with background selection, both of which occur fastest when the effective population size is small. A population bottleneck could therefore explain faster degeneration in a species such as *M*. *picta*, compared with other species. Neutral diversity has not yet been estimated in any of the fish species studied here, and effective population size estimates are not yet available. Importantly, however, this part of the process slows down after about half of the genes are lost from a fully Y-linked region, and cannot account for complete degeneration (see Fig 3 of Bachtrog 2008). The same figure illustrates that genetic hitch-hiking as advantageous mutations spread in a fully sex-linked region is slower in small populations, though this (or its combination with the ratchet, as shown in Fig 8 of the same paper) can eventually lead to complete degeneration. Assuming no back mutations (which would slow down the ratchet process), 90% loss of genes occurs in about 5 million generations, in a population with 10,000 Y chromosomes, and about 98% after 15 or 20 million generations. Complete degeneration would take longer in a larger population size.

Overall, the limited data suggest that there have probably been too few generations since the split between the *Poecilia* and *Micropoecilia* lineages for the extensive degeneration observed. However, more quantitative empirical data on degeneration and further modelling work are needed to better understand the time required for degeneration to become complete.

### Could the guppy Y have evolved recently from an X chromosome?

The evolution of the guppy Y from an X chromosome (as proposed in the second possibility above) is not implausible. First, turnovers in sex-determining genes are well documented in several groups of fish. Among species related to those discussed here, the platyfish sex-determining locus is on the homologue of the guppy chromosome 20 [38], and the more distantly related genus *Oryzias* is well known for turnovers [39], which have also been documented in cichlid fish [40], which are more distantly related still [41]. Second, the housefly offers a precedent for movement of a male-determining factor onto the X chromosome [42,43]. Mobile sex-determining regions are also known from other organisms, including Salmonid fish [44].

Turnovers are, however, thought to be less likely to occur when a species has a highly degenerated Y or W chromosome, because they may lead to low fitness homozygotes for these chromosomes being generated [45]. The proposed change in the lineage ancestral to the guppy might thus seem implausible. However, a turnover that involves a new sex-determining factor on the same chromosome, as proposed here, does not generate such homozygotes. Any slight advantage to the new system can therefore lead to a turnover [45]. A possible advantage (in an XY system, such as that in the ancestor proposed here) is that loss of genes from a degenerated Y may reduce the fitness of heterozygous males, so that the evolution of a new sex-determining factor on the X might be favoured. This is similar to the advantage proposed for repeated turnovers driven by degeneration after sex-linked regions appear [46–48].

Loss of the ancestral Y chromosome would clearly be prevented if it carried genes essential for male fertility or viability, and this has been suggested as an explanation for the long-term evolutionary stability of sex chromosomes in groups such as mammals and birds [45]. As the *M*. *picta* fully Y-linked region appears to carry very few genes, it may not carry such male function genes. Another characteristic that might make a system like that in *M*. *picta* unlikely to undergo a turnover event is that it has evolved dosage compensation of sex-linked genes [19]. If a male-determining factor appears on an autosome or an X chromosome and creates a new male heterogametic system, with loss of the ancestral Y, males in the new population will have two X chromosomes. If the dosage compensation mechanism involves higher expression of X-linked genes in males, these “XX males” might experience a deleterious high expression levels of these genes (potentially higher than in females, which will presumably have evolved to close to optimal levels). However, the mechanism controlling dosage compensation need not respond to the phenotypic sex. If instead, it responds to X chromosome dosage, the expression level could be the same in the new male genotype as in females (and in the ancestral XY genotype with hyper-expression from the X). Although *M*. *picta* has clearly not evolved an X0 sex-determining system, with the X-autosome balance controlling development of the individual as phenotypically male or female, dosage compensation might be controlled in this way.

A final potential barrier to the turnover proposed here is that the conditions for a turnover become more restrictive if sexually antagonistic genes have accumulated on the ancestral Y chromosomes [49]. However, as these authors point out, evolution of sex-limited expression of such genes on the ancestral sex chromosomes reduces any sexual conflict and allows SA alleles to become fixed, rather than remaining polymorphic, increasing the probability that a new sex-determining factor in a different genome region can invade. The absence of Y-linked coloration polymorphisms in *M*. *picta*, unlike the guppy and *M*. *parae*, suggests that this barrier may also not apply to a system like that in *M*. *picta*, though clearly the ancestral state is unknown with respect to this characteristic. Overall, the evidence from *M*. *picta* suggests that several factors expected to inhibit turnovers may not apply.

### How did the guppy and M. picta Ys evolve?

If the ancestor of the guppy and *M*. *picta* had a Y that was already highly degenerated before the split between *Poecilia* and *Micropoecilia*, a duplication of a pre-existing autosomal or sex-linked gene could have been involved, or a new maleness factor could have arisen [50]. Both types of turnover are known in fish (reviewed in refs. [39,51]). Unlike these cases, which were detected because the XY pairs are not homologous in the different species, the guppy sex chromosome pair has not changed, implying that an X-linked gene mutated, or arose by a duplication from another genome region, as in the medaka, *O*. *latipes*. In the guppy, such a duplication could have originated on an autosome or the ancestral X or Y. For example, an ancestral Y-linked male-determining factor might be superseded by a mutation of a gene that was present on the ancestral X, that conferred a male-determining action. If the ancestral Y did not carry a male-determining factor, and the ancestor had a balance system, a mutation in an X-linked gene is more plausible than duplication of or mutation in a Y-linked gene. A duplication might have led to suppressed expression of an X-linked gene that represses male functions in “normal” females, and thus created a male phenotype. Duplications with phenotypic effects that arise by repressing the expression of the progenitor gene are documented in several organisms, and is termed “co-suppression”, first discovered in transgenic plants and animals [52,53]. A similar phenomenon occurs in sex-determining systems of plants in the family Salicaceae [54–56].

**Fig 4:**
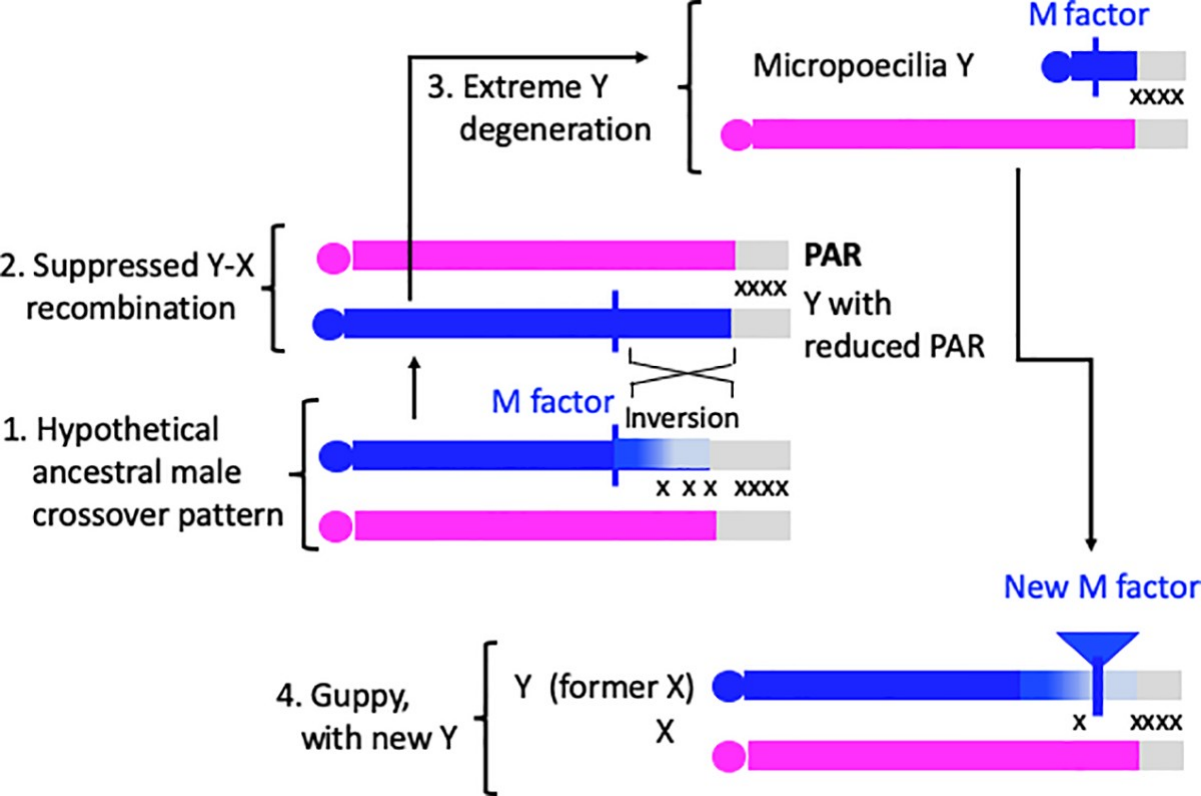
Diagram of hypothesis 2 for the evolution of the guppy Y chromosome. This hypothesis involves the appearance of a new maleness (M) factor on an X chromosome like that in *M*. *picta*. The diagram shows 4 proposed stages, indicated by numbers, from a hypothetical ancestral state (numbered 1) to the guppy state (4). Regions where crossovers occur are indicated by ‘x’ symbols, with high and low densities of symbols indicating high or low crossover rates, respectively. The ancestral state might rarely have undergone crossovers, indicated by ‘x’ symbols in a small distal part of the centromere-proximal end of the chromosome pair. A completely non-recombining Y-linked region, like that in extant *M*. *picta* males, could have evolved by an inversion (stage 2, see text), followed by complete genetic degeneration (stage 3). Possible sources of a new maleness factor in the guppy are discussed in the text. If phenotypic males rarely have crossovers in the part of the chromosome to the left of the maleness factor, this region would become closely linked with maleness. In the case of a duplication, a completely Y-linked region might also be created (expanding the guppy Y, compared with the X), but the centromere-proximal part of the chromosome could still occasionally recombine with the X, preventing genetic degeneration.

It is not known what mechanism stopped crossing over between the Y and X of *M*. *picta* and *M*. *parae*, but we speculate that, if chiasmata in the ancestral species were localised to the chromosome termini, as in the guppy [3], even a small Y chromosome inversion might have prevented crossing over between the XY pair, as diagrammed in Fig 4. An inversion could explain the complete complete sex linkage in *M*. *picta* of some genes in the guppy LG12 terminal region, PAR1 (as the rearrangement’s terminal boundary would determine the PAR boundary in stage 2 of Fig 4). However, loss of recombination in this region probably occurred recently, after recombination was suppressed across most of LG12 in *M*. *picta*, as two genes are in a non-recombining region in this species, but not in the guppy or *M*. *parae* (Table 1). Furthermore, Y-X divergence (based on intron sequences in the *zer1* gene whose sizes identify Y-versus X-linked sequences), is only 0.6%, so the PAR boundary probably changed recently in *M*. *picta*, similarly to changes detected in mammals. Finally, if a sexually dimorphic recombination pattern (heterochiasmy) was already established in a species ancestral to *M*. *picta* and *P*. *reticulata*, it would offer a simple explanation of how the ancestral X chromosome could have evolved into a Y in stage 4 in Fig 4 (possibility 1 described in the Introduction section).

The proposed appearance of a maleness factor on the X during the evolution of the guppy Y resembles events proposed in Muscid flies [42]. In some Dipteran flies, the maleness factor can appear on different chromosomes [57], including the X [43,58]. In Dipterans, crossing over does not occur in male meiosis, so that such a maleness factor immediately causes the recipient chromosome to be transmitted without recombination with the X. In the guppy, crossover patterns are sexually dimorphic, with crossovers strongly localised to the chromosome termini in males [3]. If male meiosis in the ancestor was similar, as our results suggest is true in the close outgroup of the species studied here, the platyfish, *Xiphophorus maculatus* [17], a new Y could arise in a manner similar to that in the house fly. However, Muscid fly sex chromosome turnovers appear to involve a fusion with a physically small ancestral chromosome (Muller element F) that carried an ancient sex-determining factor and is missing as an independent chromosome from such species, rather than appearance of a new maleness factor [42]. The guppy sex-determining factor is currently unknown, but a fusion scenario seems unlikely, because the male genome assembly [6] provides no evidence that any part of the XY pair is derived by fusion with, or transposition from, another chromosome, and no large region is present on the guppy XY pair but absent from either the *M*. *picta* X [26] or from the guppy Y [6]. It was recently suggested that the guppy sex-determining region arose by a duplication of a chromosome 9 region [59], but a careful examination did not support this, and suggested that assembly error could be involved [11].

It is also unlikely that the guppy Y-linked male-determining factor could have arisen by an X-Y crossover event. Such events occasionally occur in male guppies [11,15,60]. However, if the ancestral Y was degenerated, like the *M*. *picta* or *M*. *parae* Y, this implies complete lack of recombination, as a small recombination rate prevents degeneration [46].

A final possibility is that a mutation or duplication occurred on the ancestral X. Small duplications have been detected in the guppy sex chromosome pair, including in the LG12 region assembled near 20 Mb [6,11]. However, these this might simply be sequences that are linked to the male-determining locus, and not the proposed new maleness factor.

## Conclusions

Overall, we conclude that the guppy’s sex chromosomes have very different evolutionary ages: its X is an old-established X chromosome, but its Y is probably much younger. If the ancestral Y no longer carried a sex-determining gene, and all essential male function genes were carried on other chromosome(s), and a balance system of sex-determination had evolved, as suggested above, the X could have acquired a new male determining gene. Changes can occur from XY to ZW involving the same homomorphic sex chromosome pair chromosome [45], and have been documented in two frog species [61–64], and in the platyfish [65], and we argue above that X to Y transitions are also possible. Changes to a new location on the same chromosome may not be as rare as appears from the current literature, as such changes are much less easily detectable than a change that leads to a different chromosome becoming a sex chromosome. When the heterogamety is unchanged, a turnover will be particularly difficult to detect, but fine-scale genetic mapping has revealed a case in the pufferfish genus *Takifugu* [66]. However, the guppy case is different in that the change involved loss of a degenerated Y chromosome, rather than simply a change from one sex-determining gene to another on the same chromosome.

We cannot exclude the possibility of sex chromosome turnover events in the guppy lineage (after the split from the closer outgroup, *Micropoecilia*) that first shifted the sex determining locus to another chromosome, before LG12 again acquired the same sex determining locus, or evolved a different one. Turnovers are not unlikely in these species, and the sex determining locus of an outgroup to both the guppy and *Micropoecilia* lineages, *X*. *maculatus*, is on another chromosome, the homologue of the guppy LG20. In the absence of closer outgroups, this remains a formal alternative to the change diagrammed as possibility 2 in Fig 1. However, we argued above that, when the ancestor has a highly degenerated Y chromosome, changes involving the same chromosome are more likely than ones that move the sex-determining region to a different chromosome.

Moreover, the hypothesis proposed for the guppy may also explain other puzzling situations, including three species of the vole genus *Ellobius* (whose related species are generally XY, with a standard Eutherian mammal Y chromosome with strong genetic degeneration). Cytological observations suggested that both sexes have two X chromosomes, while the ancestral Y has been lost [67]; the situation therefore resembles stage 4 in Fig 4 above. It has recently been shown that the two X chromosomes in male *E*. *tancrei* differ in several properties, and that one X is male-specific, and differs distinctively from the Xs found in females [68]. This may have involved previous translocation of a pre-existing Y-linked genes to the ancestral X, maintaining male fertility functions despite Y chromosome loss [69].

The possibility that a chromosome carrying a sex-determining gene could evolve from its homologue without involving the translocation of a pre-existing male-determining region or factor could also explain the otherwise puzzling ‘non-canonical’ evolution of the W in Lepidoptera, where ZW systems have evolved from an ancestral Z0 state, yet the Z chromosomes of the different systems are homologues and carry similar sets of genes [70]. These observations suggest that the Lepidopteran W is younger than the Z. Meiosis in female Lepidoptera is achiasmate, and therefore a turnover event where the Z acquired a femaleness factor, instantly becoming a W chromosome, is possible, similar to the event proposed above for the creation of a new Y in the guppy. However, the putatively derived W chromosomes of the ZW Lepidopteran system, is shared across highly diverged species, implying that the W is much older than the guppy Y, consistent with their highly genetically degenerated state.

## Methods

### Ethics statement

The University of Edinburgh School of Biological Sciences ethics committee, SBS Ethics, reviewed and approved this research (*Ethics Assessment* ***Reference***: **dcharles-0001**).

### Fish samples, DNA extraction

*M*. *picta* samples were collected by L Yong, at Cunipia River, part of the Caroni swamp, Trinidad (10° 36 ’ 18.89 “N, 61° 25 ’ 28.462 “W), and in several other Trinidad rivers (S1 Table) by Cameron Ghalambor. A mixed natural population of *M*. *picta* and *M*. *parae* was sampled at Pronkweg road, Commewijne District, Suriname (GPS coordinates N 0712221, E 0659682). Males of the two species can be distinguished by their coloration phenotypes with reasonable accuracy [71], but females are difficult to distinguish. We therefore used several molecular markers which were found to be diagnostic for the two species (the microsatellite AG179, and intron size variants of the pseudo-autosomal genes *fpgs* and *zer1* (see S4 and S5 Tables**)**. Genomic DNA for microsatellite genotyping, genotyping of intron length variants, and for high-throughput genotyping (see below), was extracted using the Echolution Tissue DNA Kit (BioEcho, Germany). *M*. *bifurca* samples from Suriname were provided by Dr. David Reznick. The identities of all *Micropoecilia* samples were verified by sequencing their cytochrome oxidase genes, which show synonymous site divergence of at least 12% (and the sequences obtained all exceeded 437 bp).

The samples were collected under permit number 150/19 from the Ministry of Agriculture, Animal Husbandry and Fisheries of Suriname, and under an un-numbered permit from the Fisheries Division of the Department of Agriculture, Land and Fisheries of the government of Trinidad and Tobago. The study protocols were reviewed and approved by the University of Edinburgh School of Biological Sciences (see https://apps.bio.ed.ac.uk/ethrev/#/view/dcharles/1).

Microsatellite genotyping (using primers listed in S2 Table), together with initial inference of gene loss in *M*. *picta* and *M*. *parae* are described in Bergero et al. [18], which describes coverage data obtained by a high-throughput genotyping (SeqSNP) experiment. The experiment included 10 *M*. *picta* individuals of each sex, and was carried out by LGC Genomics (LGC Genomics GmbH, Ostendstrale 25, 12459 Berlin, Germany, www.lgcgroup.com/genomics); it yielded counts of the numbers of sequences obtained for each targeted region. The regions targeted were selected from within coding sequences, with about 50 bp of sequence flanking each region also being coding sequence, in order to maximise the chance that the sequence would amplify in diverse populations, and to minimise the representation of repetitive sequences. The SNPs and their locations in the guppy genome assembly are listed in a file deposited in Dryad (see above). Sequences were included only if all individuals yielded a genotype, and the mean count for the targeted site exceeded 10 per individual (usually greatly in excess of this threshold); one targeted sequence was excluded because of an exceedingly high count, suggesting a repetitive sequence. Overall, 1,338/1,749 (77%) of the autosomal coding regions targetted yielded sequences satisfying our criteria that all *M*. *picta* individuals yielded an allele count, and 411/491 of the LG12 regions (84%). The high success rate supports the expectation that nucleotide divergence between *M*. *picta* and the guppy is low (see next section and the Results section). New analyses of coverage, based on complete genome sequences, are described below.

### Estimating divergence between Poecilia and Micropoecilia sequences

As no reliably assembled genome is available, to estimate sequence divergence between *M*. *picta* and other species, we used whole genome sequences obtained from Cameron Ghalambor as raw read data (see S1 Methods 1). The names of all populations, and the sample sizes are in S1 Table. The methods used for analysing these genome sequences are described in detail in the S1 Methods text.

To obtain coding sequences from these data, an extended bioinformatics protocol was enacted using a reference-guided local assembly approach similar in principle to software such as SRAssembler and the SHORE/SUPERLOCAS low-coverage resequencing pipeline (S1 Methods 1). Because of the low depth of coverage, we used *de novo* assembled reads that mapped to genes in the *P*. *reticulata* reference genome sequence, as previous analyses of a small number of nuclear genes had shown that sequence divergence between *M*. *picta* and *P*. *reticulata* is not high [72]; for synonymous sites, the sequences available in GenBank yielded a divergence value of about 7%, and divergence based on all site types of about 2.3% (S3B Table). S1 Methods 2 describes the methods used for obtaining the coding sequences.

The between-species divergence estimates used data from *M*. *picta* females from a population sampled from a high salinity (10–19 PPT) region of the Caroni river, Trinidad (population name CAR_H). Coverage for each female was low, with a mean of 13.9 million reads, or 1.4x based on the size of the *P*. *reticulata* genome, but the pooled data from the thirteen females with high coverage and reliable sexing represented 180.6 million reads, or 18.5x coverage. The alignments for each individual gene were analysed using MEGA v7 [73], using the complete deletion option. We estimated synonymous and non-synonymous divergence, and divergence for all site types, between the species included (K_s_, K_a_ and K_all_sites_ and the sequence lengths are given in S3 Table). The MEGA files will are available in Dryad.

### Analysis of coverage in M. picta males and females

The low coverage genome sequences of our hundreds of *M*. *picta* samples were from individuals whose genomes were sequenced were of unknown sexes. We therefore used the genome sequence data to determine the sexes of the sampled individuals, based on the hemizygosity of most *M*. *picta* X-linked genes in males (S1 Methods 3, see also S2 Fig). S1 Methods 4 describes how the coverage data were used to search for *M*. *picta* LG12 genes with consistently diploid male/female coverage ratios, and to test for genes that might have additional Y-linked copies, as indicated by M/F ratios > 1.4. We also examined the coverage results for possible duplications onto the *M*. *picta* Y (S3A Fig and S3B Fig).

### Genetic and population genetic analyses in P. reticulata and in Micropoecilia species

We used two approaches to test for partial sex-linkage of markers that are assembled in the guppy sex chromosome terminal region (which are not hemizygous in in *M*. *picta* males, see the main text), genetic mapping and genotyping individuals sampled from natural populations.

First, we mapped two genes in a *P*. *reticulata* family whose parents were from a high-predation locality in the Quare river., family QHPG5 of ref. [3]. Both parents of this family were genotyped previously, and a genetic map was estimated for several microsatellite markers and numerous SNPs, distributed across LG12. For genetic mapping, we developed markers in two terminal region genes, *zer1* (at position 26,208,835 bp, encoding a *zer1* homologue protein), and *fpgs* (at position 26,286,204, which encodes a folylpolyglutamate synthase protein, mitochondrial isoform X2). The total estimated physical length of the guppy chromosome 12 is 26.5 Mb, and both these genes are distal to the most distal fully sex-linked marker in the guppy genetic maps estimated from the QHPG5 family and other families [3,11]. The *zer1* gene was mapped using a length variant in intron 10, and *fpgs* using SNPs and length difference variants for intron 4.

The *zer1* and *fpgs* genes, along with microsatellite markers used to determine individuals’ sexes [18], were also genotyped in samples of fish from natural populations, and in families, from *M*. *picta* and *M*. *parae*. The numbers of genes analysed was limited because several genes and microsatellite markers from the 1-Mb terminal region of the guppy sex chromosome assembly failed to amplify in *M*. *picta*, or yielded multiple bands, and could not be analysed.

## Supporting information

S1 Methods Methods 1: *M*. *picta* whole genome sequencing and coding sequence assembly. **Methods 2: Estimating divergence between *Poecilia* and *Micropoecilia* sequences**. **Methods 3: Protocol for sexing *M*. *picta* individuals**. **Methods 4: Searching for *M*. *picta* LG12 genes with consistently diploid coverage ratios.**
(DOCX)

S1 Table List of *M*. *picta* population samples used for analyses of coverage and heterozygosity, and the numbers of individuals that could be reliably sexed. (XLSX)

S2 Table Genetic markers used in *M*. *picta* and *M*. *parae*. A. Chromosome 12 markers used for determining hemizygosity in *M*. *picta* and *M*. *parae* males. B. Microsatellites from other chromosomes were used to determine paternity in *M*. *picta* and *M*. *parae* families and to infer heterochiasmy in *M*. *picta*.
(XLSX)

S3 Table Estimated divergence values between the guppy, *Poecilia reticulata*, and other species, including *M*. *picta*, *M*. *parae*, *Xiphophorus maculatus* (the platyfish) and *Gambusia affinis*, for multiple genes. The top part shows synonymous site divergence (Ks), non-synonymous site divergence (Ka) and divergence estimated using all site types, based on nuclear genes from multiple guppy automosomes (column F, labelled “LG” for linkage group). Ka/Ks values are also shown; most genes have values suggesting selective constraint, although two of the 22 genes (ENSPRET00000002334.1 on LG2, and ENSPRET00000011682 on LG4, appear to be evolving neutrally. Chromosome 2 of the guppy is a fused chromosome, and the genes analysed were from the arm that carries homologues of genes on *X*. *maculatus* LG7. The lower part of column Q shows Ks values between *M*. *picta* and *M*. *parae*, for 7 genes whose sequences were obtained by Pollux et al. (2014 The evolution of the placenta drives a shift in sexual selection in livebearing fish. Nature 513: 233–236. doi: 10.1038/nature13451). The Ks estimates are plotted in Fig 3.
(XLSX)

S4 Table Evidence that guppy LG12 genes behave as sex-linked in *M*. *picta*. The table shows genotypes in *M*. *picta* for variants in loci on the guppy sex chromosome, LG12 (microsatellite markers and variants in the zer1 and fpgs genes). Sibships were harvested from gravid females sampled in the wild from natural populations in Trinidad (family numbers T1 to T7) and Suriname (families S1 to S3). The positions of the markers genotyped in the female guppy genome assembly are indicated below the marker names. Tissue from the female parent was used to genotype the mother. Using the microsatellite loci, the progeny were inferred to be male when all loci with variants in the family have only a single one of the dam’s X-linked alleles, consistent with hemizygosity of the X chromosome in males. Although the data for these loci are limited, the genotypes were consistent with sex linkage in all families in which they were informative. The X-linked alleles of the fathers at each locus were then deduced from the genotypes of female progeny. These data indicated that many sibships were sired by multiple males; the sires of most female progeny could be assigned. The dam and inferred sire genotypes are shown first for each family, with the observed progeny genotypes below. For the zer1 genes, males were not hemizygous, allowing us to determine the sire for some male progeny. Two progeny each had genotypes for one microsatellite marker (highighted in yellow) that do not completely correspond to our inferences based on the other markers; in both cases, the allele sizes were only 2 bp different from another allele in the family, and could have been mis-classified.
(XLSX)

S5 Table Genotypes of two guppy PAR genes in *M*. *picta* and *M*. *parae* fish from natural populations. Most *M*. *picta* individuals were sampled from a population in Trinidad (shown in the table on the left), and *M*. *parae* were sampled from a population in Suriname, together with a few *M*. *picta* individuals (right hand table). The positions in the guppy female assembly are shown under the gene names.
(XLSX)

S6 Table Evidence for a degenerated Y chromosome in *Micropoecilia bifurca*. Microsatellite markers were selected using the guppy (*Poecilia reticulata*) female or male assembly to find markers in positions spanning the whole chromosome. Three markers showed no variants in the female sample. The other six "informative" markers had two or more different alleles in females (and both homozygotes and heterozygotes were seen), but they all appear to be hemizygous in males (different alleles were found in the males also, but all males that could be genotyped appeared to be homozygotes; at least 13 males support this conclusion for each informative marker). The identities of the *M*. *bifurca* samples studied were verified using *cox1* sequences, as for *M*. *picta* and *M*. *parae* (see S5 Table).
(XLSX)

S7 Table Empirical data on the relationship between synonymous site divergence (*K*_s_ values) and genetic degeneration. (XLSX)

S1 Fig Divergence estimates between the guppy and its close relatives. (TIFF)

S2 Fig Example of the analysis for determining the sexes of individuals from a population, using the X/A ratios based on genes assembled on the guppy LG12 and an autosome, LG22. (TIFF)

S3 Fig Upset plots showing the results of coverage analyses of LG12 sequences that are diploid in males, versus either haploid (indicating loss of recognizable sequence of a gene) or indicating Y-linked duplications. (TIFF)

S4 Fig Dot plot showing the relationship between guppy LG12 sequences and sequences in the platyfish, *Xiphophorus maculatus*, whose homologous chromosome is Xm8. Genetic mapping in male meiosis shows that in the guppy the PAR is at the right-hand end of LG12, indicating that the centromere of this telocentric chromosome is at the left. In the platyfish, the order of sequences is reversed, so that sequences corresponding to the guppy PAR are assigned low number on the y axis. The red lines show GC content, and the low value in the boxed region indicates unusually high AT, consistent with the clear evidence that this region has high repetitive content.
(TIFF)

S5 Fig Dot plot of the relationship between the two different assemblies of the guppy LG12. The x axis shows the female assembly, and the male assembly positions are shown on the y axis. sequences and sequences in the platyfish, *Xiphophorus maculatus*, whose homologous chromosome is Xm8. Genetic mapping in male meiosis shows that in the guppy the PAR is at the right-hand end of LG12, indicating that the centromere if this telocentric chromosome is at the left. In the platyfish, the order of sequences is reversed, so that sequences corresponding to the guppy PAR are assigned low number on the y axis.
(TIFF)

S6 Fig The relationship between sequences in the assembly of chromosome 1 of the platyfish, *Xiphophorus maculatus*, and their guppy homologues. The homologue of this chromosome is the guppy LG7, and the box indicates the region where a small set of genes are assembled on LG12 in the female guppy assembly. In *M*. *picta*, these genes are part of a 6.3 Mb contig whose other genes correspond to guppy LG7 genes (see main text).
(TIFF)

S7 Fig Male/female coverage ratios of genes (y axis value) and presence of exons in the *M*. *picta* contigs corresponding to the region of the guppy LG12 distal to 20 Mb in the female assembly. The x axis shows the guppy female assembly positions in base pairs. Each gene’s coverage value is shown as a grey dot, and when more exons were detected in a small physical distance the dots are darker in colour. The physical extents of the *M*. *picta* contigs are shown below the coverage plot. Changes from each contig to its adjacent contig are indicated by changes in the colour, and the dots indicate individual exons. It can be seen that some regions have low gene density (the dots representing coverage are grey, not black), and these also tend to correspond to gaps between contigs. However, in the most terminal region, with the highest M/F ratios, most exons are present as single copies in *M*. *picta*.
(TIFF)

S8 Fig Male and female coverage in the part of the *M*. *picta* LG12 corresponding to the region shown in S7 Fig), and coverage values relative to the values for an autosome, LG15. The red horizontal lines indicate ratios of 1, 0.5 and 0. The values were estimated from genome sequence data on 166 and 157 females, from samples from 11 natural populations (S1 Table). The relative coverage values were estimated for each sex separately for each population, as different populations might yield different results in the sequencing, and the overall means for the two sexes were then calculated. In the region between 20 and 25 Mb the sex difference in coverage (M/F coverage ratio) is similar to the value of 0.5 for the rest of LG12 (see Fig 2 and S7 Fig), but the relative (M/A and F/A) values are lower in both sexes). In the most distal region, where M/F coverage values are variable, and often high in one sex or the other. 40 of the 64 genes have lower relative coverage in males than females, ten higher in males, and 14 have similar coverage in both sexes. Note that our analyses excluded low coverage and low quality sequences (as described in S1 Methods 1).
(TIFF)
