# Supplementary material for: How did the guppy Y chromosome evolve?
Source: PLoS Genet. 2021 Aug 9;17(8):e1009704. doi: 10.1371/journal.pgen.1009704 (PMC8376059; doi:10.1371/journal.pgen.1009704)
Supplement: S1 Methods — Methods 2: Estimating divergence between Poecilia and Micropoecilia sequences. Methods 3: Protocol for sexing M. picta individuals. Methods 4: Searching for M. picta LG12 genes with consistently diploid coverage ratios. (DOCX) [file pgen.1009704.s001.docx]

**S1 methods**

**Contents**

**Methods 1:** *M. picta* whole genome sequencing and coding sequence assembly……………………………………………………………………………………………………………. Page 1

**Methods 2:** Estimating divergence between *Poecilia* and *Micropoecilia* sequences…………………………………………………………………………………………………………. Page 2

**Methods 3:** Protocol for sexing *M. picta* individuals…………………. Page 3

## *Preparing the reference genome and annotation* ……….………………….……. Page 3

*Population quality control* ………………………………………………………….………….. Page 5

**Methods 4:** Searching for *M. picta* LG12 genes with consistently diploid coverage ratios …………………………………………………………………………………………………… Page 5

# **Appendix: Scripts for the analyses in the sections above** (written by Chay Graham)

## 1-fastqc.sh…………………………………………………………………………………………………… Page 11

## 2-trimmomatic.sh………………………………………………………………………………………. Page 12

3-analysis…………………………………………………..………………………………………………………… Page 14

**Methods 1:**

***M. picta*** **whole genome sequencing and coding sequence assembly**

Our analyses used whole *M. picta* genome sequences obtained from Cameron Ghalambor (Colorado State University), as raw read data. The complete sample, from multiple natural populations, is described in Supplementary Table S1. Fin clips taken at the river were preserved in ethanol at -20^0^F. DNA was extracted using a Qiagen Blood/Tissue kit with the default protocol. Sequencing was completed by Novogene on an Illumina HiSeq X10, producing 2x150bp paired-end reads with an expected 300bp insert, using standard Illumina adapter and primer libraries. The data for each sequenced individual’s raw read library, including the sequences used in other analyses described below, are split into forward and reverse libraries.

Demultiplexed raw reads were first assessed with FastQC v0.11.8, and pre-processed with Trimmomatic v0.39’s ILLUMINACLIP function to remove adapters as listed in the adapters.fa file of BBMap v38.63 (ILLUMINACLIP:adapters.fa:2:30:10). Further quality control was applied with Trimmomatic to remove trailing start or end N stretches, or stretches where base quality was lower than 3 (LEADING: 3 TRAILING: 3), and then a sliding window which clipped reads if quality fell below mean 15 over a 4 base window was applied (SLIDINGWINDOW:4:15). After these processing steps, reads less than 108 bp in length were discarded (MINLEN: 108).

The remaining paired reads were mapped to the *P. reticulata* reference genome using BWA v0.7.17, utilising the bwa mem tool with the default settings. The resulting alignment was converted to bam format, indexed, sorted and filtered with SAMtools 1.9, removing unmapped reads, optical and PCR duplicates, and reads with mapping quality < 30. The number of reads in trimmed libraries, the number of mapping reads, and number of mapping reads that passed quality control checks were all taken for each individual to monitor library sizes and recall. Recall was high, averaging around 0.75, with some 2.7 billion reads mapping at high quality from the original 3.6 billion, from more than 300 individuals. Male recall did not differ from general recall.

The script used for trimming is in the appendix: **2-trimmomatic.sh**.

**Methods 2: Estimating divergence between *Poecilia* and *Micropoecilia* sequences**

To find coding regions for estimating inter-species divergence, we used data from thirteen *M. picta* females (sexed as described in **Methods 3** below) sampled from a population in a high salinity (10-19 PPT) region of the Caroni river, Trinidad (population CAR-H in Supplementary Table S1) and sequenced as described above. Coverage for each female was low, with a mean of 13.9 million reads, or 1.4x using the size of the *P. reticulata* genome as an estimate for the *M. picta* genome size. The maximum coverage among these individuals was 5.4x. When the thirteen females were pooled, they represented 180.6 million reads, or 18.5x coverage. The low coverage nature of our data contra-indicated whole genome assembly; we instead *de novo* assembled reads that mapped to genes in the *P. reticulata* reference genome sequence, as *M. picta* and *P. reticulata* sequences are expected to be similar (Supplementary Table S2 and Figure S1). File manipulation made extensive use of SAMtools, BEDTools v2.26.0 and seqkit v0.10.1 in pipelines available on request from the authors. Ensembl annotations for whole-genome cds in guppy, platyfish and mosquitofish were used throughout these steps. Recall was high, with over 140 million reads mapping at high quality.

We chose two genes from each *P. reticulata* chromosome. For a given *P. reticulata* coding sequence, we took the genomic coordinates of the surrounding region, and extracted *M. picta* reads which mapped to this region from the merged BAM alignment, then converted *M. picta* reads to paired FASTQ files, along with the original *P. reticulata* reference sequence for the region. Reads from *M. picta* were de novo assembled with Minia v3.2.1, with a kmer value of 51 and relaxed settings (-tip-len-topo-kmult 1, -tip-rctc-cutoff 20) that enabled reads mapping to local areas to be used in assembly, even when coverage was low in some of the 13 females.

Once assembled, the resulting contigs were aligned to the *P. reticulata* reference and scaffolded using the PAGIT pipeline (VM installation v1.64), using the tools ABACAS and IMAGE. First, ABACAS (implemented with nucmer) was used to order *M. picta* contigs along the *P. reticulata* reference genome assembly (excluding contigs shorter than 300bp with the parameter: -l 300). The mapped and unmapped contigs were then concatenated and passed to IMAGE as scaffolds. Scaffolding with IMAGE used stringent settings (-smalt_minScore 135) to ensure that only long, high-matching reads were used in contig merging, and other settings were customized for our data (-vel_ins_len 300, -kmer 51). Iterations were continued until extension or merging stopped and the subsequent iteration had no merging. The contigs2scaffold.pl script was then used for this iteration, with a minimum contig size of 300, and used to mark gaps with 6 N characters. Numbers of IMAGE iterations are shown in Supplementary Table S2A.

The longest single contig from the above scaffolding process was aligned with four other sequences: the *P. reticulata* reference region, and the cds in *P. reticulata* {Künstner, 2017 #14120}, *X. maculatus* {Schartl, 2013 #12615} and *G. affinis* {Hoffberg, 2018 #14924}*.* Alignment was completed in two stages, first using MAFFT g-ins-i with appropriate settings to handle antisense gene regions (--adjustdirection). This produced a multifasta alignment with the three cds aligned in gap-separated blocks spaced over the *P. reticulata* and *M. picta* gene region, which were taken to be the coding regions. The alignments were then manually adjusted in a text editor, and bases in the *M. picta* and *P. reticulata* larger gene regions without an alignment to any of the reference cds were removed to produce putative coding sequences. The *P. reticulata* region served as a control to identify where differences in annotated cds were present in the larger *P. reticulata* genomic context. Whilst this approach is biased towards producing a *M. picta* fragment that is similar to the *P. reticulata* reference, confidence in the approach is supported by the recovery of large *M. picta* fragments that include sequence that is present in *X. maculatus* and *G. affinis* but entirely missing from the *P. reticulata* reference, suggesting that our approach also finds sequences in *M. picta* that are either deleted or poorly assembled in *P. reticulata*.

**Methods 3: Protocol for sexing *M. picta* individuals**

The samples whose genomes were sequenced were of unknown sexes. We therefore used the information that the *M. picta* X-linked genes are mostly hemizygous in males as the basis for a sexing procedure.

# Mapping and quality control were done as follows, for each population sample.

## *Preparing the reference genome and annotation*

The genome of was indexed with samtools. The Ensembl gff3 annotation was parsed to extract several bed files containing coordinates of genes, exons and 10kb windows to use in the protocol. The reference genome was indexed for use with bwa mem. Reads for each individual were trimmed, mapped and filtered as described in **Methods 1**, using bwa mem with the default settings. We additionally counted numbers of reads at each stage of filtration (used for the later library normalisation stage) and enacted coverage analysis with bedtools using each of our region files. Coverage results were robust with any of the bed files, and we report results based on genes. The following scripts were used to analyse each population sample:

samtools view -q 30 -b /scratch/cgraham/alignment_$1/"$line"_aln-pe-nodup.bam > /scratch/cgraham/alignment_$1/"$line"_aln-pe-finished.bam

### re-index the finished reads.

samtools index /scratch/cgraham/alignment_$1/"$line"_aln-pe-finished.bam

## optional filter for reads that haven't failed vendor or machine checks and, re-indexing

### samtools view -F 0x0200 -b "$line"_aln-pe-finished.bam > "$line"_aln-pe-vendorcheck.bam

### samtools index "$line"_aln-pe-vendorcheck.bam

### compare this to the finished alignment from the stage before (to likely observe no difference), or use the 'vendorcheck.bam' file for the further steps.

######## counting numbers of reads at each stage of filtration (key for library normalisation later)

### count all reads, and print the number to the first line of a new text file

samtools view -c --threads 60 /scratch/cgraham/alignment_$1/''$line''_aln-pe.bam -o /scratch/cgraham/alignment_$1/''$line''_reads.txt

### count mapping reads, and append this number to the reads text file on a new line

samtools view -c --threads 60 /scratch/cgraham/alignment_$1/''$line''_aln-pe-mapping.bam >> /scratch/cgraham/alignment_$1/''$line''_reads.txt

### count mapping reads that pass all QC stages and are included in the final mapping, and append this number to the reads text file on a new line

samtools view -c --threads 60 /scratch/cgraham/alignment_$1/''$line''_aln-pe-finished.bam >> /scratch/cgraham/alignment_$1/''$line''_reads.txt

########## coverage calling

### the general strategy is to use: bedtools coverage -mean -a [.bed file with regions of interest] -b [bam file with mapping] > [.txt file with coverages]. The output will be a tab-separated file with the following columns: accession, start, end, mean depth of region. Each row will correspond to each row in the .bed file input, which specifies the regions of interest, and rows with no depth will also be recorded. We will exploit the fact that for a given .bed input file, the output always has the same number of rows.

### call coverage for each .bed file of interest

bedtools coverage -mean -a reticulatagenes.bed -b /scratch/cgraham/alignment_$1/''$line''_aln-pe-finished.bam > /scratch/cgraham/alignment_$1/''$line''_genecovdepth.txt

bedtools coverage -mean -a reticulataexonsensembl.bed -b /scratch/cgraham/alignment_$1/''$line''_aln-pe-finished.bam > /scratch/cgraham/alignment_$1/''$line''_exoniccovdepth.txt

bedtools coverage -mean -a reticulatawindows_1kb.bed -b /scratch/cgraham/alignment_$1/''$line''_aln-pe-finished.bam > /scratch/cgraham/alignment_$1/''$line''_1kb_windowdepth.txt

### for each output, gzip the results to make transferring faster downstream (especially important for granular windows).

gzip /scratch/cgraham/alignment_$1/''$line''_genecovdepth.txt

gzip /scratch/cgraham/alignment_$1/''$line''_reads.txt

gzip /scratch/cgraham/alignment_$1/''$line''_exoniccovdepth.txt

gzip /scratch/cgraham/alignment_$1/''$line''_1kb_windowdepth.txt

########## end while loop! Supply as an input to the while loop the text file as a first argument.

done < $1

*Population quality control*

Before analysing results, the read counting results were inspected to identify populations whose libraries yielded outliers. We checked whether, within and between populations:

- library sizes are reasonably high, to avoid small libraries
- mapping recall is similar for each library
- mapping recall with QC is similar for each library

Libraries with >1,000,000 reads after mapping and QC were considered potentially usable. Any results with recall less than two thirds, or recall with QC reads below two thirds, or where there was a difference between the two of more than ~25-30% were noted, and results checked to ensure that they were robust to the exclusion of these libraries.

Normalisation used the total library size of each individual after trimming, but before mapping and filtering (i.e. unprocessed read counts). For populations where some individuals have poor recall but still retain many reads, we used mapping and QC (i.e. 'finished') read counts instead of total reads as a normaliser, to avoid contaminants. Finally, the coverage depths of genes from LG12 vs. LG15 were used to determine individuals’ sexes, using the ratio of depths (denoted X/A). Sexing (see Supplementary Figure S2) was done using R and Linux command line scripts. A full R script is given in the appendix as **3-analysis.R**, with transitions to Linux noted.

**Methods 4: Searching for *M. picta* LG12 genes with consistently diploid coverage ratios**

These results were the basis for the coverage analysis of individual LG12 genes, to obtain M/F ratios (Figure 2 of the main text). We used over 300 individuals taken from 12 populations for this stage of analysis, detailed in Supplementary Table 1. LG12 genes with M/F > 0.8 were noted and considered candidates for diploid coverage, while genes with lower coverage were considered to be likely to be hemizygous. Two scaffolds that were unplaced in the guppy female genome assembly {Fraser, 2020 #15196} (NW_007615023 and NW_007615031) were analysed along with LG12 because they include genetic markers that have been mapped to LG12 {Charlesworth, 2020 #15281}. We also noted putatively autosomal genes with M/F > 1.4, as these are candidates for genes that additionally have Y-linked copies. Assuming that there are two autosomal copies in both males and females, we expect the ratio to be (2+y)/2, where *y* is the number of Y-linked copies. Values consistently close to 1.5 across populations suggest a single added Y copy, and other values close to multiples of 0.5 would indicate other copy numbers.

To obtain as many LG12 genes with consistently diploid coverage ratios as candidate genes that have not been lost from the *M. picta* Y chromosome, before merging the results from different populations, we tested whether genes in our list for this chromosome are shared between populations. The results were visualised as UPSET plots (Supplementary Figure S3). For each population, the M/F scores were calculated for each gene, and we listed all genes with putative Y copies in both the categories just defined. We calculated intersection sizes for all possible combinations of gene lists (after removing duplicates). We additionally produced M/F ratios based on pooling all individuals across all populations; results were robust after repeating this with the pooled values added as a 'population'.

The input file for UpSetR was created by the following commands

## run function on our list of results produced as described above

intersectionnamed<-overlap(listinput)

## collect the names of each intersection in UpSetR-ready format
namesofintersections<-names(intersectionnamed)

## collect sizes of intersections

## initialise empty vector

sizesofintersections<-c()

## iterate from the first entry 1 to the maximum number of combinations. For 12 different populations (11 plus a pooled set) this is 4,095 combinations.

for (k in 1:4095){

sizesofintersections<-c(sizesofintersections,length(intersectionnamed[[k]]))}

### write the output

write.table(as.data.frame(cbind(namesofintersections, as.numeric(sizesofintersections))), file = "Intersection_sizes.txt", quote = T, sep = "=", row.names = F)

This output was then formatted to make it ready for UpSetR, by removing the first line and the quotation marks around the numbers, and replacing the end of line ($) quote (") with commas (including for the last line):

cat Intersection_sizes.txt | sed 's/\"\=\"/\"\ \=\ /g' | sed 's/\"$/,/g' > Intersection_sizes_parsed.txt

#### the fromExpression() function converts a vector of expressions as above into an object that can be plotted by the upset() function

library(UpSetR)

input_asexpression <-fromExpression(input)

png(filename = "UPSET_all.png", width = 4320, height = 1200)

upset(input_asexpression,
#### nintersects as NA will plot all results

nintersects = NA,
#### nsets should be the number of sets, treating the pooled set as a set if included

nsets = 12,

order.by = "freq",

decreasing = F,

mb.ratio = c(0.6, 0.4),

number.angles = 0,

text.scale = 1.1,

point.size = 2.8,

line.size = 1

)

dev.off()

### For a simpler plot (Supplementary Figure 3B), using the selection of results that satisfied our criteria, and removing the pooled set, the following code was used

inputoriginal <- c(

CAP_F = 55,

CAP_H = 72,

CAP_L = 89,

CAP_S = 82,

CAR_F = 66,

CAR_H = 86,

CAR_L = 80,

CAR_S = 90,

CUN_F = 84,

CUN_L = 90,

CUN_S = 97,

#Pooled = 97,

"CAP_F&CAP_H&CAP_L&CAP_S&CAR_F&CAR_H&CAR_L&CAR_S&CUN_F" = 30,

"CAP_F&CAP_H&CAP_L&CAP_S&CAR_F&CAR_H&CAR_L&CAR_S&CUN_F&CUN_L" = 30,

"CAP_F&CAP_H&CAP_L&CAP_S&CAR_F&CAR_H&CAR_L&CAR_S&CUN_F&CUN_L&CUN_S" = 30,

"CAP_F&CAP_H&CAP_L&CAP_S&CAR_F&CAR_H&CAR_L&CAR_S&CUN_F&CUN_S" = 30,

"CAP_F&CAP_H&CAP_L&CAP_S&CAR_F&CAR_H&CAR_L&CAR_S&CUN_L&CUN_S" = 31,

"CAP_F&CAP_H&CAP_L&CAP_S&CAR_F&CAR_H&CAR_L&CUN_F&CUN_L&CUN_S" = 31,

"CAP_F&CAP_H&CAP_L&CAP_S&CAR_F&CAR_H&CAR_S&CUN_F" = 30,

"CAP_F&CAP_H&CAP_L&CAP_S&CAR_F&CAR_H&CAR_S&CUN_F&CUN_L" = 30,

"CAP_F&CAP_H&CAP_L&CAP_S&CAR_F&CAR_H&CAR_S&CUN_F&CUN_L&CUN_S" = 30,

"CAP_F&CAP_H&CAP_L&CAP_S&CAR_F&CAR_H&CAR_S&CUN_F&CUN_S" = 30,

"CAP_F&CAP_H&CAP_L&CAP_S&CAR_F&CAR_L&CAR_S&CUN_F" = 30,

"CAP_F&CAP_H&CAP_L&CAP_S&CAR_F&CAR_L&CAR_S&CUN_F&CUN_L" = 30,

"CAP_F&CAP_H&CAP_L&CAP_S&CAR_F&CAR_L&CAR_S&CUN_F&CUN_L&CUN_S" = 30,

"CAP_F&CAP_H&CAP_L&CAP_S&CAR_F&CAR_L&CAR_S&CUN_F&CUN_S" = 30,

"CAP_F&CAP_H&CAP_L&CAP_S&CAR_F&CAR_S&CUN_F" = 30,

"CAP_F&CAP_H&CAP_L&CAP_S&CAR_F&CAR_S&CUN_F&CUN_L" = 30,

"CAP_F&CAP_H&CAP_L&CAP_S&CAR_F&CAR_S&CUN_F&CUN_L&CUN_S" = 30,

"CAP_F&CAP_H&CAP_L&CAP_S&CAR_F&CAR_S&CUN_F&CUN_S" = 30,

"CAP_F&CAP_H&CAP_L&CAP_S&CAR_H&CAR_L&CAR_S&CUN_F" = 30,

"CAP_F&CAP_H&CAP_L&CAP_S&CAR_H&CAR_L&CAR_S&CUN_F&CUN_L" = 30,

"CAP_F&CAP_H&CAP_L&CAP_S&CAR_H&CAR_L&CAR_S&CUN_F&CUN_S" = 30,

"CAP_F&CAP_H&CAP_L&CAP_S&CAR_H&CAR_S&CUN_F&CUN_L" = 30,

"CAP_F&CAP_H&CAP_L&CAP_S&CAR_H&CAR_S&CUN_F&CUN_L&CUN_S" = 30,

"CAP_F&CAP_H&CAP_L&CAP_S&CAR_H&CAR_S&CUN_F&CUN_S" = 30,

"CAP_F&CAP_H&CAP_L&CAR_F&CAR_H&CAR_L&CAR_S&CUN_F&CUN_L" = 30,

"CAP_F&CAP_H&CAP_L&CAR_F&CAR_H&CAR_L&CAR_S&CUN_F&CUN_S" = 30,

"CAP_F&CAP_H&CAP_L&CAR_F&CAR_H&CAR_S&CUN_F&CUN_L&CUN_S" = 30,

"CAP_F&CAP_H&CAP_L&CAR_S&CUN_F" = 30,

"CAP_F&CAP_H&CAR_S" = 31,

"CAP_F&CAP_H&CAR_S&CUN_F" = 30,

"CAP_F&CAP_H&CUN_F" = 31,

"CAP_F&CAP_S&CAR_S&CUN_F" = 30,

"CAP_F&CAR_H&CAR_L&CAR_S&CUN_F&CUN_L" = 30,

"CAP_F&CAR_H&CAR_L&CAR_S&CUN_F&CUN_S" = 30,

"CAP_H&CAP_L&CAP_S&CAR_F&CAR_H&CAR_L&CAR_S&CUN_F&CUN_L&CUN_S" = 43,

"CAP_H&CAP_L&CAP_S&CAR_F&CAR_H&CAR_L&CAR_S&CUN_L&CUN_S" = 44,

"CAP_H&CAP_L&CAP_S&CAR_F&CAR_H&CAR_L&CUN_F&CUN_L&CUN_S" = 45,

"CAP_H&CAP_L&CAP_S&CAR_H&CAR_L&CAR_S&CUN_F&CUN_L&CUN_S" = 60,

"CAP_H&CAP_L&CAP_S&CAR_H&CAR_L&CUN_F&CUN_L&CUN_S" = 62,

"CAP_H&CAP_L&CAR_H&CAR_L&CAR_S&CUN_F&CUN_L&CUN_S" = 62,

"CAP_L&CAP_S&CAR_H&CAR_L&CAR_S&CUN_L&CUN_S" = 69,

"CAP_L&CAP_H&CAP_S" = 64,

"CAP_L&CAP_H&CAP_S&CAP_F" = 32,

"CAP_L&CAP_S&CAR_F&CAR_H&CAR_L&CAR_S&CUN_F&CUN_L&CUN_S" = 45,

"CAP_L&CAP_S&CAR_H&CAR_L&CAR_S&CUN_F&CUN_L&CUN_S" = 65,

"CAP_L&CAP_S&CAR_H&CAR_L&CAR_S&CUN_L&CUN_S" = 69,

"CAP_L&CAR_H" = 81,

"CAP_L&CAR_H&CAR_L&CAR_S&CUN_L&CUN_S" = 73,

"CAP_L&CAR_H&CAR_L&CUN_L&CUN_S" = 75,

"CAP_L&CAR_H&CUN_L&CUN_S" = 78,

"CAP_L&CUN_L&CUN_S" = 81,

"CAP_L&CUN_S" = 85,

"CAR_H&CAP_H" = 69,

"CAR_H&CUN_L" = 80,

"CAR_H&CUN_S" = 83,

"CAR_L&CAP_L&_CUN_L" = 76,

"CAR_L&CAP_L&_CUN_L&CAR_H&CAP_H" = 66,

"CAR_L&CAP_L&_CUN_L&CAR_H&CAP_H&CAR_S&CAP_S&CUN_S" = 61,

"CAR_L&CAR_H&CAR_S" = 76,

"CAR_L&CAR_H&CAR_S&CAR_F" = 48,

"CAR_S&CAP_S&CUN_S" = 72,

"CUN_F&CAP_F&CAR_F" = 34,

"CUN_L&CUN_S" = 85,

"CUN_L&CUN_S&CUN_F" = 74

)

# Plot with combined syntax

upset(fromExpression(inputoriginal),

nintersects = NA,

nsets = 11,

order.by = "freq",

decreasing = F,

mb.ratio = c(0.6, 0.4),

number.angles = 0,

text.scale = 1.1,

point.size = 2.8,

line.size = 1

)

We then visualised our results as UPSET plots. We first displayed all results, to help identify sets of samples that lack reliable results for some genes, and to select sets for inclusion in the downstream analysis. We then selected combinations that represented the overall trends in set sizes. These initial results led to exclusion from the final analysis of the least informative (smallest) population samples, which often lack genes with data, whereas this is rarer for the larger samples. We considered genes robustly evidenced if they had the requisite M/F ratios in all non-excluded populations. All but one gene (located on LG8) classified this way had non-zero read counts in both sexes from all non-excluded populations, supporting their validity. Results for candidate LG12 genes with diploid coverage (M/F > 0.8, shown in Figure S3A) excluded 4 populations, and those for candidate putatively autosomal genes with additional Y-linked copies (M/F > 1.4, shown in Figure S3B) excluded 5 populations). Information from excluded populations was retained in our spreadsheets, and in overall pooled M/F ratios using all individuals, which were considered throughout our evaluation of the results.

The final coverage results were combined across all populations except for the few with small samples that lacked reliable data for many genes. Figure 2 in the main text shows the M/F gene coverage for genes assembled on LG12 (top) and LG15 (bottom).

# Appendix: Scripts for the analyses described above (written by Chay Graham)

## 1-fastqc.sh

#!/bin/bash

#$ -N fastqc

#$ -cwd

#$ -l h_vmem=16G

### usage: sh 1-fastqc.sh [file which is a line-separated list of fastq file names to operate on]

### note: this can be adapted for cluster running

source /ceph/software/conda/etc/profile.d/conda.sh

conda activate /ceph/users/cgraham/.conda/envs/fastqc

### give java memory in the virtual environment

java -Xmx16G

### initialise while loop

### run fastqc on default settings and specify output name as default

while read -r line; do

fastqc /data/charlesworth/guppy/analyses/trimmedpubpicta/$line -o /data/charlesworth/guppy/analyses/public_picta_fqc/trimmed/

done < $1

## 2-trimmomatic.sh

#!/bin/bash

#$ -N trimmo

#$ -cwd

#$ -pe smp 60

#$ -l h_vmem=32G

date +"%m-%d-%y"

date +"%T"

###ulimit -S -n 4096

### usage: sh 2-trimmomatic.sh [file name with list of prefixes for fastq files]

source /ceph/software/conda/etc/profile.d/conda.sh

conda activate /ceph/users/cgraham/.conda/envs/trimmomatic

### clean up scratch space

rm -r /scratch/cgraham

#rm -r /scratch/cgraham/trimmed

### initialise scratch space

mkdir /scratch/cgraham

mkdir /scratch/cgraham/trimmed_$1

### iterate over each prefix file

while read -r line; do

trimmomatic PE -phred33 -threads 60 ''$line''_1.fq.gz ''$line''_2.fq.gz -baseout /scratch/cgraham/trimmed_$1/''$line''_trimmed.fq.gz ILLUMINACLIP:adapters.fa:2:30:10 LEADING:3 TRAILING:3 SLIDINGWINDOW:4:15 MINLEN:108;

done < $1

### print date after trimming

date +"%m-%d-%y"

date +"%T"

### copy results

mkdir /data/charlesworth/guppy/analyses/ghalambor/trimmed_$1

rsync -r /scratch/cgraham/trimmed_$1 /data/charlesworth/guppy/analyses/ghalambor/trimmed_$1

### print date and time after completion

date +"%m-%d-%y"

date +"%T"

## 3-analysis.R

### example is for CAP_S population

### change working directory to folder with coverage files

setwd("/Users/chaygraham/Documents/Research/Edinburgh/Working2/Ghalambor/pops/CAP_S/genecov/")

### in Linux command line, set up a file called 'files' which has each line as file name of the readouts from bedtools coverage

### throughout, 'exons' is used as a placeholder for 'regions of interest', which in this case are genes

### read in genes of interest in the format accession-start-end

exons <- read.delim("reticulatagenesreworkrestrict.bed", header = F)

### read in list of files to operate over

files<-read.delim("files", header=F)

### convert file names to a vector

files<-files$V1

### convert factors to character strings

files<-as.character(files)

### initialise large table of all individuals

allindivs <- exons

#### for each file, read in the coverage data, and select only the fourth column containing the coverage results, and add this to a growing table

for (i in files)

{indiv <- read.delim(i, header = F)

allindivs<- cbind(allindivs, indiv[,4])}

### calculate mean depth summed across all sites of interest for each individual

# at LG12

means<- colMeans(allindivs[which(allindivs$V1 =="NC_024342.1"),-c(1,2,3)])

# at LG15
means_auto<- colMeans(allindivs[which(allindivs$V1 =="NC_024345.1"),-c(1,2,3)])

# calculate X/A ratio using LG15

means_ratio<- colMeans(allindivs[which(allindivs$V1=="NC_024342.1"),-c(1,2,3)])/colMeans(allindivs[which(allindivs$V1=="NC_024345.1"),-c(1,2,3)])

### write means to files

write.table(means, file = "means")

write.table(means_auto, file = "means_auto")

write.table(means_ratio, file="means_ratio")

### at this point, devise a naming scheme based on X/A results

### rename column headers in large table of all results. This will be different for each population or set of individuals analysed.

colnames(allindivs) <- c("Accession", "Start", "End", "m1",

"m2",

"f1",

"m3",

"m4",

"ll1",

"m5",

"f2",

"f3",

"m6",

"m7",

"m8",

"f4",

"m9",

"f5",

"f6",

"f7",

"f8",

"f9",

"ll2",

"ll3",

"f10",

"f11",

"f12",

"m10")

### initialise new table to correct depth scores

allindivs_corr <- allindivs

### for normalisation, ensure you have a line separated list of read counts, or another library size proxy, that will be used in normalisation, with lines in order of the individuals to be corrected

### read in normalisation counts

reads <- read.delim("reads.txt", header=F)

### convert to vector of numbers

reads <- as.vector(reads$V1)

### for each individual, divide each site's depths by the library size provided

for (j in 4:length(colnames(allindivs_corr))){

allindivs_corr[,j] <- allindivs_corr[,j]/reads[j-3]}

### combine males, summing at each site

males_corr <- (allindivs_corr$m1+allindivs_corr$m2+allindivs_corr$m3+allindivs_corr$m4+allindivs_corr$m5+allindivs_corr$m6+allindivs_corr$m7+allindivs_corr$m8+allindivs_corr$m9+allindivs_corr$m10)

### combine females, summing at each site

females_corr <- (allindivs_corr$f1+allindivs_corr$f2+allindivs_corr$f3+allindivs_corr$f4+allindivs_corr$f5+allindivs_corr$f6+allindivs_corr$f7+allindivs_corr$f8+allindivs_corr$f9+allindivs_corr$f10+allindivs_corr$f11+allindivs_corr$f12)

### create an adjusted M/F ratio at each site with the number of individuals used included to scale the results

mf_corr_adj <- (males_corr/10)/(females_corr/12)

### create a vector of unique chromosomes/scaffolds/accession headers

chroms<-as.character(unique(exons$V1))

### plot results for each individual at LG12, with the plot named after the individual

for(j in 4:length(colnames(allindivs_corr))){

png(paste(colnames(allindivs_corr[j]), ".png", sep=""), width = 900, height = 600)

plot(x = allindivs$Start[which(allindivs$Accession=="NC_024342.1")], y = allindivs[which(allindivs$Accession=="NC_024342.1"),j], pch=19, cex=.4, xlab="Position (bp)", main=colnames(allindivs_corr[j]), ylab="Mean depth", ylim=c(0,4))

dev.off()}

### create a function to plot a particular chromosome/scaffold/accession across the scaled results from all individuals

plot1<-function(numb){

png(filename = paste(chroms[numb], ".png", sep = ''), width = 900, height = 500)

plot(allindivs_corr$Start[which(allindivs_corr$Accession==chroms[numb])], mf_corr_adj[which(allindivs_corr$Accession==chroms[numb])], pch=19, cex=.3, xlab="Position (bp)", ylim=c(0,4), ylab="M/F", main=chroms[numb])

dev.off()

}

### use this plotting function across all chromosomes, creating plots named after the chromosome/accession

for (k in 1:length(chroms)) {

plot1(k)

}

#### format results and write them as tables to file

allindivs_corr_full <- cbind(allindivs_corr, mf_corr_adj)

### write all results

write.table(x=allindivs_corr_full, file="full_results.txt", quote = F, sep = "\t",row.names = T, col.names = T)

### select results > 1.4 MF

write.table(x=allindivs_corr_full[which(allindivs_corr_full$mf_corr_adj >1.4),], file="1.4 results", quote=F, sep="\t", row.names=T, col.names = T)

### select results > 0.8 MF and on LG12

write.table(x=allindivs_corr_full[which(allindivs_corr_full$mf_corr_adj >0.8 & allindivs_corr_full$Accession =="NC_024342.1"),], file="0.8 sex chr results", quote=F, sep="\t", row.names=T, col.names = T)

### select results with > 1.4 MF

CAP_S_onepointfour<- allindivs_corr_full[which(allindivs_corr_full$mf_corr_adj >1.4),]

### read in all genes with gff3 annotation

allgenes<-read.delim("Poecilia_reticulata.Guppy_female_1.0_MT.genes.gff3", header=F)

### annotate results

onepointfourannotated<-merge(x = allgenes, y = CAP_S_onepointfour, by.x = "V4", by.y = "Start")

### could optionally include dplyr package command 'distinct()' to remove duplicate rows

write.table(onepointfourannotated, file = "mfonepointfour", quote = F, sep = "\t", row.names = T, col.names = T)

### selecting M/F > 0.8 on LG12

CAP_S_diploid <- allindivs_corr_full[which(allindivs_corr_full$mf_corr_adj >0.80 & allindivs_corr_full$Accession =="NC_024342.1"),]

### annotation produced by merging commands (use dplyr or Linux uniq command to reduce duplicates)

doublesannotated<-merge(x = allgenes, y = CAP_S_diploid, by.x = "V4", by.y = "Start")

write.table(doublesannotated, file = "diploidsexchr", quote = F, sep = "\t", row.names = T, col.names = T)

### store results to variables named after the population for collection

CAP_S_males <- males_corr

CAP_S_females <-females_corr
