# Supplementary figures and images for: How did the guppy Y chromosome evolve?
Source: PLoS Genet. 2021 Aug 9;17(8):e1009704. doi: 10.1371/journal.pgen.1009704 (PMC8376059; doi:10.1371/journal.pgen.1009704)

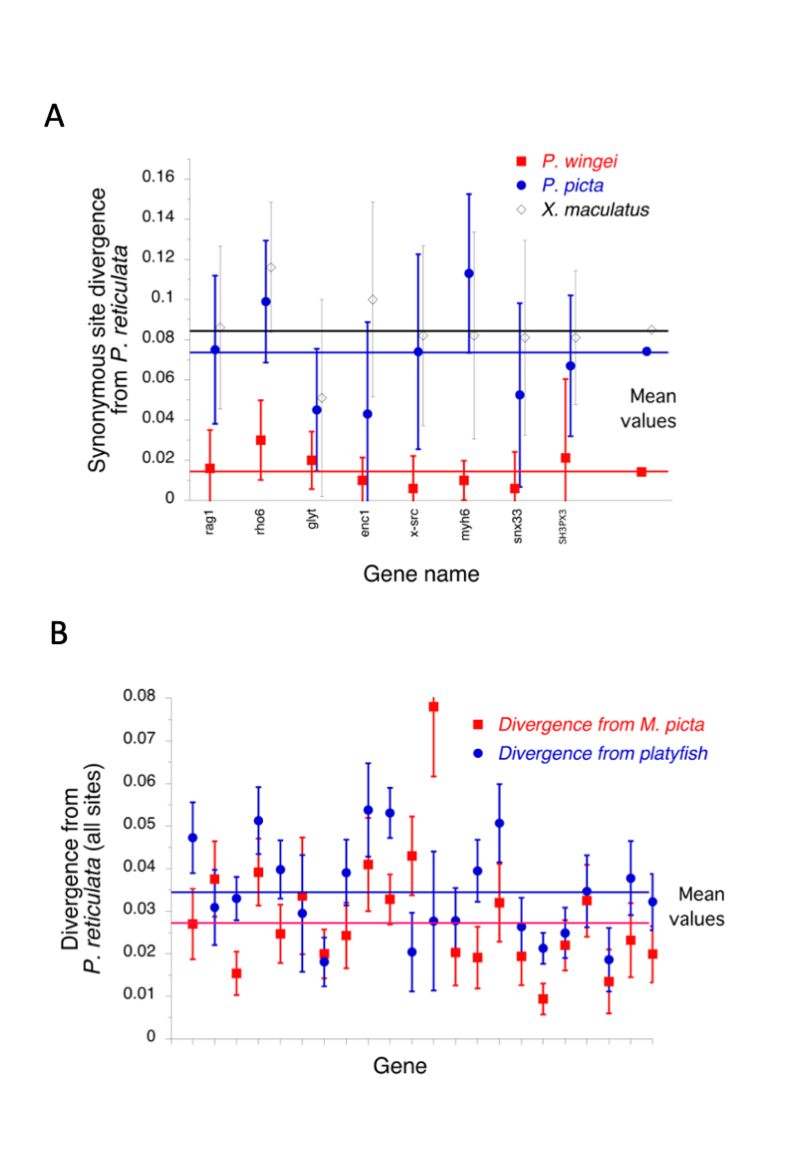

Supplement: S1 Fig — (TIFF) [file pgen.1009704.s009.tiff]

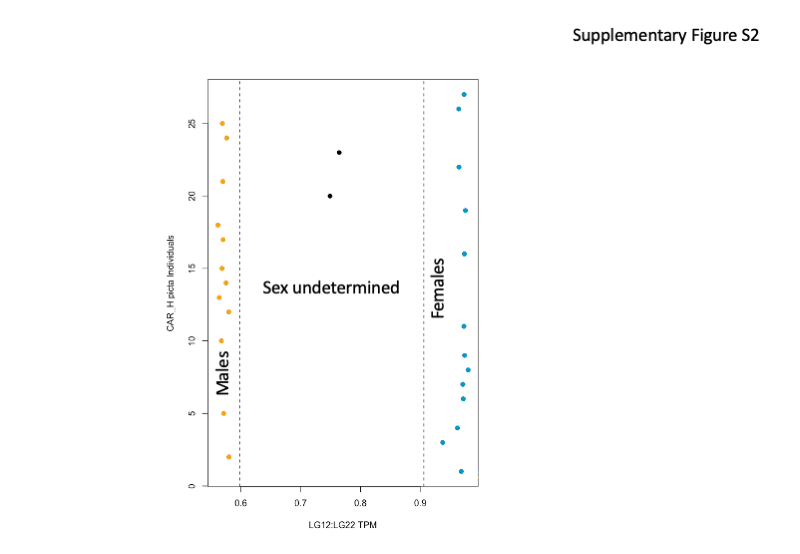

Supplement: S2 Fig — (TIFF) [file pgen.1009704.s010.tiff]

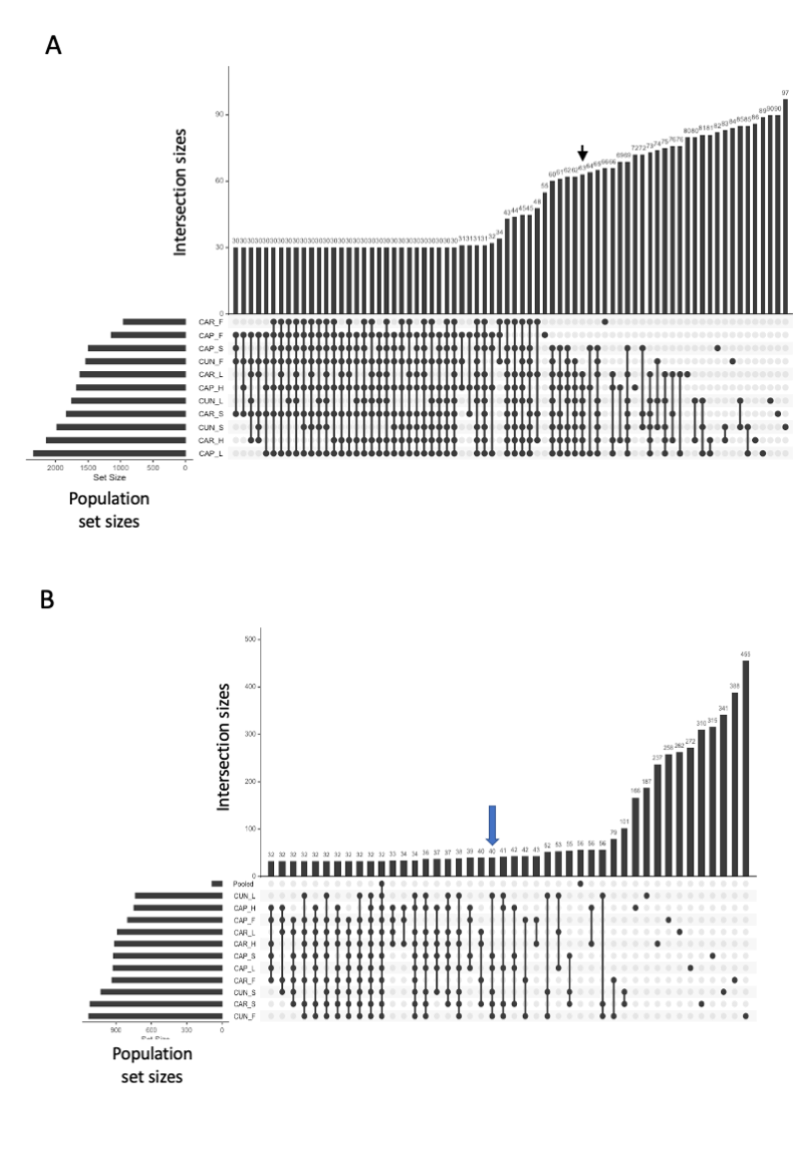

Supplement: S3 Fig — (TIFF) [file pgen.1009704.s011.tiff]

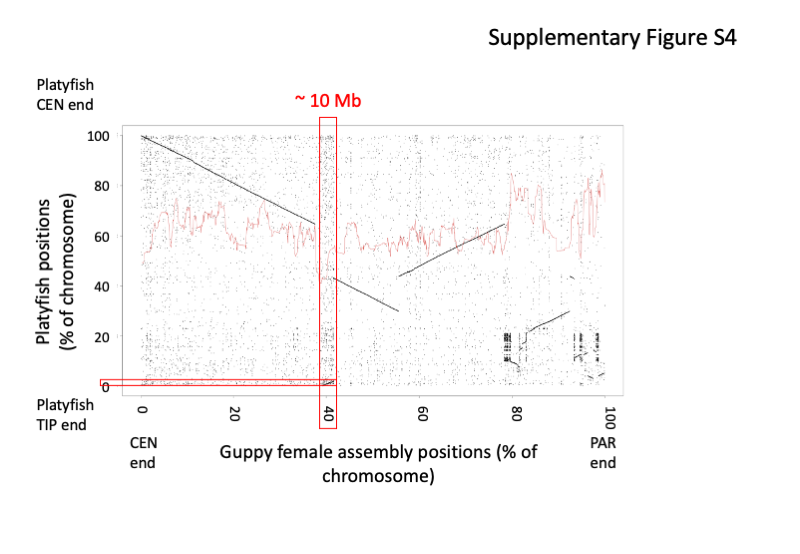

Supplement: S4 Fig — Genetic mapping in male meiosis shows that in the guppy the PAR is at the right-hand end of LG12, indicating that the centromere of this telocentric chromosome is at the left. In the platyfish, the order of sequences is reversed, so that sequences corresponding to the guppy PAR are assigned low number on the y axis. The red lines show GC content, and the low value in the boxed region indicates unusually high AT, consistent with the clear evidence that this region has high repetitive content. (TIFF) [file pgen.1009704.s012.tiff]

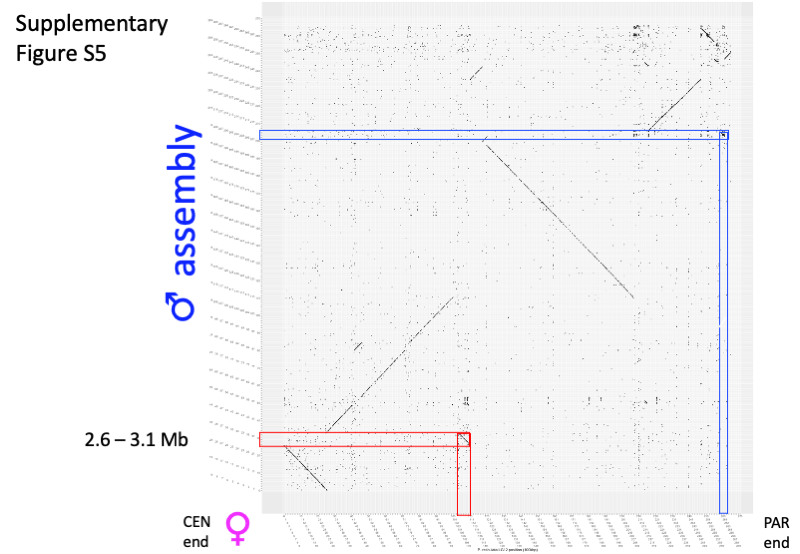

Supplement: S5 Fig — The x axis shows the female assembly, and the male assembly positions are shown on the y axis. sequences and sequences in the platyfish, Xiphophorus maculatus, whose homologous chromosome is Xm8. Genetic mapping in male meiosis shows that in the guppy the PAR is at the right-hand end of LG12, indicating that the centromere if this telocentric chromosome is at the left. In the platyfish, the order of sequences is reversed, so that sequences corresponding to the guppy PAR are assigned low number on the y axis. (TIFF) [file pgen.1009704.s013.tiff]

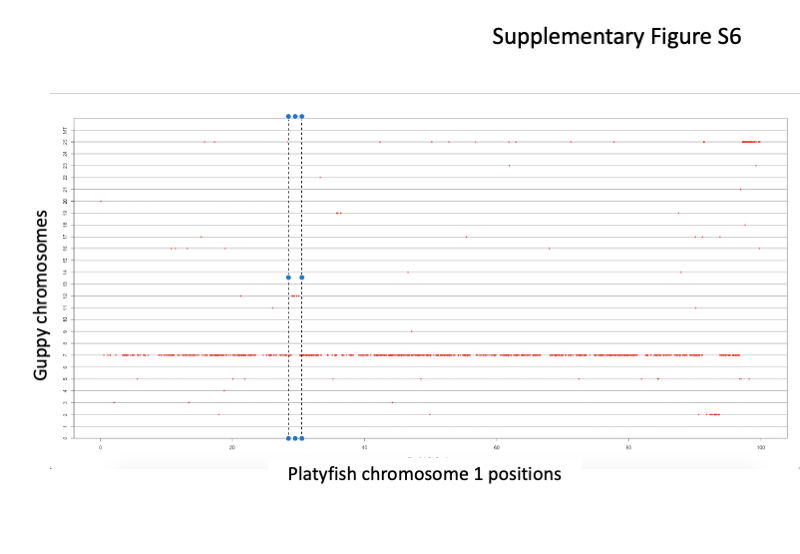

Supplement: S6 Fig — The homologue of this chromosome is the guppy LG7, and the box indicates the region where a small set of genes are assembled on LG12 in the female guppy assembly. In M. picta, these genes are part of a 6.3 Mb contig whose other genes correspond to guppy LG7 genes (see main text). (TIFF) [file pgen.1009704.s014.tiff]

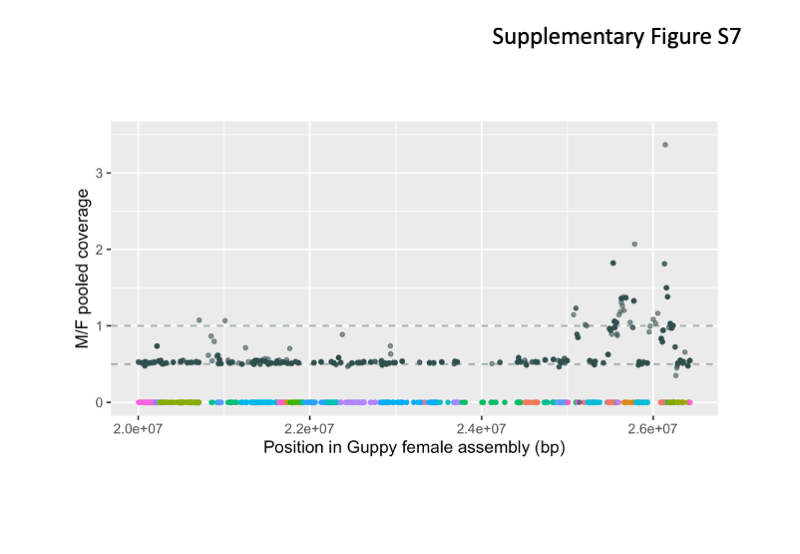

Supplement: S7 Fig — The x axis shows the guppy female assembly positions in base pairs. Each gene’s coverage value is shown as a grey dot, and when more exons were detected in a small physical distance the dots are darker in colour. The physical extents of the M. picta contigs are shown below the coverage plot. Changes from each contig to its adjacent contig are indicated by changes in the colour, and the dots indicate individual exons. It can be seen that some regions have low gene density (the dots representing coverage are grey, not black), and these also tend to correspond to gaps between contigs. However, in the most terminal region, with the highest M/F ratios, most exons are present as single copies in M. picta. (TIFF) [file pgen.1009704.s015.tiff]

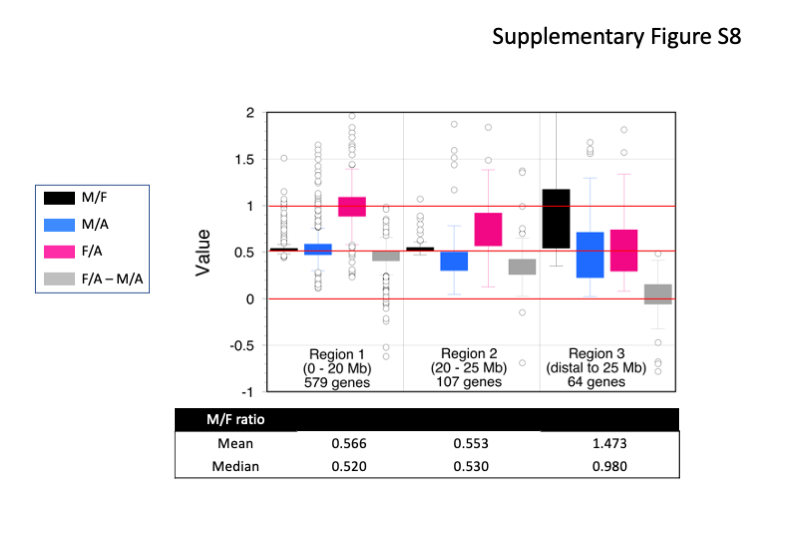

Supplement: S8 Fig — The red horizontal lines indicate ratios of 1, 0.5 and 0. The values were estimated from genome sequence data on 166 and 157 females, from samples from 11 natural populations (S1 Table). The relative coverage values were estimated for each sex separately for each population, as different populations might yield different results in the sequencing, and the overall means for the two sexes were then calculated. In the region between 20 and 25 Mb the sex difference in coverage (M/F coverage ratio) is similar to the value of 0.5 for the rest of LG12 (see Fig 2 and S7 Fig), but the relative (M/A and F/A) values are lower in both sexes). In the most distal region, where M/F coverage values are variable, and often high in one sex or the other. 40 of the 64 genes have lower relative coverage in males than females, ten higher in males, and 14 have similar coverage in both sexes. Note that our analyses excluded low coverage and low quality sequences (as described in S1 Methods 1). (TIFF) [file pgen.1009704.s016.tiff]
